# Determination of the Transport Efficiency in spICP-MS Analysis Using Conventional Sample Introduction Systems: An Interlaboratory Comparison Study

**DOI:** 10.3390/nano12040725

**Published:** 2022-02-21

**Authors:** Otmar Geiss, Ivana Bianchi, Guillaume Bucher, Eveline Verleysen, Frédéric Brassinne, Jan Mast, Katrin Loeschner, Lucas Givelet, Francesco Cubadda, Francesca Ferraris, Andrea Raggi, Francesca Iacoponi, Ruud Peters, Anna Undas, Alexandra Müller, Ann-Katrin Meinhardt, Birgit Hetzer, Volker Gräf, Antonio R. Montoro Bustos, Josefa Barrero-Moreno

**Affiliations:** 1 European Commission, Joint Research Centre (JRC), 21027 Ispra, Italy; ivana.bianchi@ec.europa.eu (I.B.); josefa.barrero@ec.europa.eu (J.B.-M.); 2 Service Commun des Laboratoires (SCL), 33608 Pessac, France; guillaume.bucher@free.fr; 3 Sciensano, Trace Elements and Nanomaterials, 1180 Brussels, Belgium; eveline.verleysen@sciensano.be (E.V.); frederic.brassinne@sciensano.be (F.B.); jan.mast@sciensano.be (J.M.); 4 Division for Food Technology, National Food Institute, Technical University of Denmark, 2800 Lyngby, Denmark; kals@food.dtu.dk (K.L.); lgiv@food.dtu.dk (L.G.); 5 Istituto Superiore di Sanità (ISS), National Institute of Health, 00161 Rome, Italy; francesco.cubadda@iss.it (F.C.); francesca.ferraris@iss.it (F.F.); andrea.raggi@iss.it (A.R.); francesca.iacoponi@iss.it (F.I.); 6 Wageningen Food Safety Research, Part of Wageningen University & Research, Business Unit Contaminants & Toxicology, 6708 Wageningen, The Netherlands; ruudj.peters@wur.nl (R.P.); anna.undas@wur.nl (A.U.); 7 Max Rubner-Institut (MRI), Federal Research Institute of Nutrition and Food, Department of Food Technology and Bioprocess Engineering, 76131 Karlsruhe, Germany; alexandra.mueller@mri.bund.de (A.M.); ann-katrin.meinhardt@mri.bund.de (A.-K.M.); birgit.hetzer@mri.bund.de (B.H.); volker.graef@mri.bund.de (V.G.); 8 National Institute of Standards and Technology (NIST), Gaithersburg, MD 20899, USA; antonio.montorobustos@nist.gov

**Keywords:** single particle ICP-MS, transport efficiency, gold nanoparticles

## Abstract

In single particle inductively coupled plasma mass spectrometry (spICP-MS), the transport efficiency is fundamental for the correct determination of both particle number concentration and size. In the present study, transport efficiency was systematically determined on three different days with six carefully characterised gold nanoparticle (AuNP) suspensions and in seven European and US expert laboratories using different ICP-MS instruments and spICP-MS software. Both particle size—(TES)—and particle frequency—(TEF)—methods were applied. The resulting transport efficiencies did not deviate much under ideal conditions. The TEF method however systematically resulted in lower transport efficiencies. The extent of this difference (0–300% rel. difference) depended largely on the choice and storage conditions of the nanoparticle suspensions used for the determination. The TES method is recommended when the principal measurement objective is particle size. If the main aim of the measurement is the determination of the particle number concentration, the TEF approach could be preferred as it might better account for particle losses in the sample introduction system.

## 1. Introduction

Due to its high sensitivity, elemental specificity and capability to simultaneously measure particle number concentration and size, single particle inductively coupled plasma mass spectrometry (spICP-MS) has become increasingly popular for metal/metal-oxide (nano)particle analysis in recent years [1,2]. In spICP-MS, as well as in ICP-MS in general, when conventional sample introduction systems are used, only a fraction of the nebulised suspension effectively reaches the plasma (1–15%) [3,4,5]. The precise determination of this fraction, which is defined as transport efficiency (η_n_), is fundamental for the correct determination of both particle number concentration and size [4,6]. An exception for sizing is the use of a nanoparticle (NP) calibration standard with the same chemical composition as the targeted NP to establish a response factor to relate signal intensity and particle mass [7]. Transport efficiency can be determined following various methods. The two most common and reliable methods, also described in the ISO Technical Specification ISO/TS 19,590 [8], are the particle size (TES) and the particle frequency (TEF) methods. Table 1 provides an overview of these two methods.

Only a few studies have been published in which both TES and TEF methods are compared. A reference study in the field of spICP-MS is the work done by Pace and co-workers [4]. In this study, the transport efficiency was measured on multiple days, using three separate methods: TES, TEF and the waste collection method. For the former two, the monodispersed citrate-stabilised 60 nm AuNP reference material (RM) from the National Institute of Standards and Technology (NIST) was used (NIST RM 8013). For each run, the particle frequency and particle size methods produced matching efficiencies while the value obtained applying the waste collection method was strongly overestimated. Givelet and co-workers [9] conducted a study in which they optimised an analytical method for the characterisation of titanium dioxide nanoparticles in food additives and pharmaceuticals. In this study, a number of parameters were assessed, among which is the transport efficiency. Determined following both the TEF and TES methods, the impact of transport efficiencies on the mean diameter and the nanofraction (i.e., fraction of particles <100 nm) of the representative test material NM-100 (titanium dioxide, anatase) was evaluated. Results varied by 18% for the mean diameter and by 34% for the nanofraction for NM-100. The authors concluded that the TEF method provided more accurate results comparing the obtained average diameters and nanoparticulate fractions with reported values from other techniques in the literature. Aznar and co-workers [10] used spICP-MS to detect, quantify and characterise the number-based particle size distribution of silver nanoparticles in different environmental samples (e.g., lake waters), aqueous samples derived from migration tests and consumer products. TEF, TES and the waste collection method were compared. Based on better interday repeatability, the authors rated the frequency approach best for the determination of the transport efficiency. Interday variations of the determined transport efficiencies can be strongly influenced by daily optimisation procedures of the ICP-MS instrument. Intraday variabilities were not provided in the study. Another recent study [11] compared spICP-MS performance with small-angle X-ray scattering spectroscopy (SAX). In this study, transport efficiency was determined following the three classical methods (TEF, TES and waste collection). The obtained values were then evaluated in terms of reproducibility and accuracy. According to these assessment criteria, the particle size method proved to be more relevant. In a study that compared two sample preparation techniques, alkaline and enzymatic treatment, for characterising nanoparticles in human placental tissue by spICP-MS, it was investigated if AuNPs and ionic Au standard solutions should be prepared in the same matrix as the digested tissue samples for determination of transport efficiency [12]. Here, similar transport efficiencies (6–8%) were determined by the TEF and TES method, independently of the used matrix. The only exception was observed for the transport efficiency (11%) determined by the TES method in the alkaline matrix. This was related to a 54% higher ICP-MS response for ionic Au in the alkaline matrix in comparison to the ultrapure water and enzyme solution. Liu and co-workers [13] conducted a study which aimed at identifying potential pitfalls and providing practical recommendations for spICP-MS analysis through evaluation with well characterised particles from NIST (NIST RM 8017). Transport efficiency was determined with NIST RM 8013. The authors found appreciable discrepancies between TES and TEF (TEF lower) and concluded that the size method yields the more robust measure of transport efficiency. Bucher and Auger [14] also reached the same conclusion when comparing TEF and TES for 60 nm gold nanoparticles. They attributed the slightly lower transport efficiency obtained by the frequency method to uncertainties of the particle number concentration in the analysed suspension.

The accuracy of measurements done with spICP-MS depends largely on the precise determination of the transport efficiency. So far, however, the various methods that can be used to determine transport efficiency and the parameters affecting its determination have not been systematically and critically evaluated. In the present study, transport efficiency was systematically determined on three different days with five commercially available and one candidate reference material AuNP-suspension, and in seven expert laboratories using different ICP-MS instruments and spICP-MS software, applying both TES and TEF methods. Parameters that may affect the determination of transport efficiency which were treated in this study were the accuracy of the AuNP size and mass concentration (including the presence of a potential ionic fraction), surface treatments (citrate and PEG), the choice of the dilution solvent of the AuNPs (ultrapure water or Na-citrate), the selection of fitting curves during the determination of TES and the possible impact of dilution. Moreover, transport efficiencies obtained using AuNPs were compared with those obtained with other types of nanoparticles, namely silver and platinum. The impact of uncertainties of transport efficiency on the particle number concentration and particle diameter of a titanium dioxide and a gold NP-containing sample was assessed as well. The results of this study are intended to complement information previously published in the literature and in the ISO technical specifications [8] and to serve as a guide for the spICP-MS community using conventional sample introduction systems.

## 2. Methods and Materials

### 2.1. Selection of Gold Nanoparticle Suspensions

A number of commercially available gold nanoparticle suspensions were selected for this study. Detailed knowledge of the number size distribution and the mass concentration of the gold nanoparticle suspensions used for the determination of transport efficiency is essential. In the past, monodispersed NIST RM 8013 with well-defined mean size, size distribution and Au mass concentration has been widely used by the spICP-MS community for the determination of both TES and TEF. However, NIST RM 8013 has been out of stock for a long time. A NIST candidate reference material (NIST CaRM), citrate stabilised AuNPs was instead included in the list of materials tested in this study. No detailed information of particle size and particle number concentration was provided for this material. In addition to the NIST CaRM, a selection of alternative commercially-available AuNP suspensions were investigated (Table 2). Selection criteria for these materials were the average size of the particles, the surface treatment and dispersant, the broadness of the number size distribution and the mass concentration of the initial non-diluted product. The diameter of the selected particles ranged approximately from 30 nm to 80 nm, which covers the size range typically selected for calibration and transport efficiency determination by the spICP-MS community. In this size range, complete atomisation and ionisation in the plasma can be assumed [11]. Most of the tested products were accompanied by product specification sheets (certificates of analysis, CoA), which included all this information. It was, however, decided to verify some of these parameters such as the gold mass concentration, the size distribution and the possible presence of dissolved or ionic Au, as their accuracy has a significant impact on the determination of the transport efficiency.

### 2.2. Qualitative Assessment of the Number Size Distribution with spICP-MS

As a first qualitative assessment before shipping the test materials to the laboratories participating in this study, the number size distribution of the tested materials was determined with spICP-MS. To this end, first the transport efficiency was determined based on the TES method, followed by re-measuring the same suspension as a sample. Tested AuNP suspensions were diluted to reach approximately 100,000 particles mL^−1^. Solutions of dissolved gold (blank and four solutions ranging from 1 µg L^−1^ to 5 µg L^−1^) were prepared by diluting the stock solutions with ultrapure water. Ionic gold standard solutions for ICP-MS (1 g L^−1^ in 5% HCl) were purchased from Sigma-Aldrich (St. Louis, MO, USA).

A Perkin Elmer NexIon 300D quadrupole ICP-MS, equipped with an SC Fast peristaltic pump, a Meinhard concentric nebuliser, a glass cyclonic spray chamber and a standard quartz torch (2.0 mm injector i.d.) operating in standard mode was used for spICP-MS analysis (Perkin Elmer, Waltham, MA, USA). Operating conditions were checked daily to achieve maximum sensitivity. For the setting of all parameters and data acquisition, the Nano Application Module of Syngistix™ software version 2.5 was used. The dwell time was set at 100 μs and the total data acquisition time at 60 s. The exact flow rate of the peristaltic pump required for the determination of the transport efficiency was measured gravimetrically daily and ranged between 0.15 mL min^−1^ and 0.18 mL min^−1^. For the determination of AuNP suspensions, the gold-197 isotope was monitored setting the mass fraction and the ionisation efficiency to 100% and density to 19.3 g cm^−3^.

### 2.3. Verification of Number Size Distribution with Electron Microscopy

The precise knowledge of the particle size is fundamental for the reliable determination of transport efficiency based on the TES method, and indirectly also for the TEF method. Bias in particle size measurement was shown to be asymmetric considering that the derived mean particle number concentration is based on the size to the inverse third power [15]. For all tested products except the NIST candidate reference material, the specification sheet included information on the average particle size. However, not all values were determined with the same analytical technique. Considering that the size characterisation of commercially-available NPs is typically limited to the analysis of only 100 NPs, leading to data that is insufficient to define the distribution and the mean diameter accurately [7], it was therefore decided to verify those values. A comprehensive verification was done with transmission electron microscopy (TEM) and with scanning electron microscopy (SEM).

#### 2.3.1. Transmission Electron Microscopy (TEM)

All samples were concentrated by bringing 500 μL of dispersion into a 1.5 mL Eppendorf vial and centrifuging at 17,500 rpm (30,184× *g*) for 30 min using a 5430R centrifuge (Eppendorf, Hamburg, Germany) equipped with rotor FA-45-24-11-HS. The supernatant (450 μL) was removed, and the pellet was re-suspended in 25 μL of ultrapure water. After 30 s of vortex stirring, the sample was brought on an Alcian blue-treated pioloform and carbon-coated, 400 mesh copper grid (Agar Scientific, Essex, England) by the grid-on-drop method [16]. For each material, a set of 10 representative images was recorded using a 120 kV Tecnai G2 Spirit TEM with BioTwin lens configuration (Thermo Fisher Scientific, Eindhoven, The Netherlands), equipped with a 4 × 4 k Eagle charge-coupled device (CCD) camera (Thermo Fisher Scientific) while using the TEM imaging and analysis (TIA) software (Version 3.2, Thermo Fisher Scientific). The magnification and the associated pixel size (i.e., the lower limit of detection (LLOD)) were determined based on the criterion of Merkus [17]. The lower limit of quantification (LLOQ) was defined as ten times the LLOD. The corresponding upper limit of quantification (ULOQ) was limited to one tenth of the image size (i.e., the upper limit of detection (ULOD)), supporting on ISO 13322-1 [18]. For the material N8151035, a magnification of 49,000 was applied. This results in a LLOD of 0.2 nm, a LLOQ of 2 nm and an ULOQ of 91.7 nm. For the other materials, a magnification of 18,500 was applied. This results in a LLID of 0.6 nm, a LLOQ of 6 nm and an ULOQ of 245 nm. For both magnifications, the useful ranges contained all detected particles. The size and shape properties of the constituent particles were estimated from the properties of their 2D projections using the ParticleSizer software [19]. For each particle, the (maximum) Feret diameter (Fmax), the minimum Feret diameter (Fmin) and the equivalent circular diameter (ECD) were determined. The raw data resulting from the image analysis was processed using an in-house Python script for calculation of descriptive statistics and plotting histograms, following ISO 9276-1 [20] guidelines for representation of results of particle size analysis. Expanded uncertainties (95%) on the measurements were determined combining repeatability and intermediate precision, estimated in top-down validation studies, with calibration and trueness uncertainties [21].

#### 2.3.2. Scanning Electron Microscopy (SEM)

The following sample preparation protocol was applied for all six AuNP suspensions: stock solutions were sonicated for 5 min in an ultrasonic bath (USC300T, VWR, Germany) and 1:5 diluted with ultrapure water (Milli-Q IQ7000, Merck KGaA, Germany). After 60 s vortex stirring, 6 µL of each sample were applied to a Si wafer (5 × 7 mm chips, Plano GmbH, Germany) by drop deposition and air-dried. Images were recorded with a FEI Quanta 250 Scanning Electron Microscope (FEI, Brno, Czech Republic) with a field emission gun (FEG) as electron source. All analyses were carried out under high vacuum conditions with an Everhart-Thornley detector with an acceleration voltage of 20 kV. To avoid large systematic deviations, the magnification of the images was chosen to be in the range of 80,000–200,000×, so that the smallest measurable nanoparticle had at least an area of 100 pixels [22]. For image processing and size measurement, the AuNP ImageJ (v1.52p) in combination with the NanoDefine Particle Sizer-Plugin were used, which enable the stack analysis of several images in one step. For stack analysis 5–8 images of each sample were chosen, which were recorded at several different positions on the Si wafer, where the particles are spread homogeneously without being stacked. However, small particle aggregates consisting of 2–8 primary particles were accepted for the size measurement process. Overall, at least 200 particles were measured for deriving a particle size distribution. The mean and median values for the minimum and the maximum Feret diameter (Fmin, Fmax) and the equivalent circular diameter (ECD) were extracted from the data output sheet and further processed with Excel/Sigmalplot (v14.0).

### 2.4. Impact of the Dispersant on the Stability of AuNP Suspensions

#### 2.4.1. Assessment of the Stability of AuNP Suspensions with Dynamic Light Scattering (DLS) Measurements at Concentrations of 3 mg L^−1^

The impact of the dispersant on the stability of AuNP suspensions was assessed by preparing working suspensions of approximately 3 mg L^−1^ for all test materials except N8151035 AuNPs. Stock concentration of this material was at least three orders of magnitudes lower than the rest of the test materials, which turned out to be below the operational range of DLS. Dilutions were prepared in both ultrapure water and 1.5 mM sodium citrate. DLS measurements of these suspensions were done at defined time intervals (0, 2, 5, 24, 48 and 120 h). Before the first measurement (t_0_), the suspensions were bath-sonicated for 5 min and vortex-mixed for 30 s. In all following measurements, samples were vortex-mixed but not sonicated. All measurements were done in triplicate and each replicate was measured three times. A Zetasizer Nano-ZS (Malvern Panalytical Ltd., Malvern, UK) was used to perform DLS measurements. The DLS settings included the automatic optimisation of the measurement conditions (measurement position/depth and attenuator). The dispersant viscosity was set at 0.8872 mPa·s (water at 25 °C).

#### 2.4.2. Assessment of the Stability of AuNP Suspensions with spICP-MS at Nominal Concentrations of 100,000 Particles mL^−1^

The impact of the dispersant on the stability of AuNP suspensions was assessed at a much lower particle number concentration using spICP-MS for the determination of the number size distribution at various time intervals. For that, working suspensions of approximately 100,000 particles mL^−1^ of two of the tested AuNP suspensions (AUCN60 and N8151035) were prepared in both ultrapure water and 1.5 mM sodium citrate. Number size distributions were determined at various time intervals. TES was determined only one at time t_0_ using test material N8151035 suspended in ultrapure water. Ionic Au-standard solution calibrants were prepared in ultrapure water. Instrumental conditions were the same as described under Section 2.2.

### 2.5. Determination of Mass Concentrations of Tested AuNP Suspensions

The exact knowledge of the mass concentration of the tested products is of fundamental importance, especially when determining the TEF. Here the dilution required to reach the particle concentration in the operational range for spICP-MS is derived from the particle number concentration in the undiluted product. This in turn is obtained by conversion through the mass concentration and the declared particle size. The mass concentration was determined by ICP-MS and inductively coupled plasma—optical emission spectrometry (ICP-OES) with and without acidic digestion.

#### 2.5.1. Determination of Mass Concentration of Non-Digested AuNP Suspensions

A calibration curve of ionic Au standard solutions was prepared in ultrapure water (blank, 10, 30, 60, 100 µg L^−1^). All solutions were prepared gravimetrically, using an intermediate solution of 10 mg L^−1^ of ionic Au. The intermediate solution was obtained by diluting a commercially available 1000 mg L^−1^ Au standard solution. AuNP working suspensions were diluted with ultrapure water to reach an Au mass concentration of 50 µg L^−1^. All ionic Au standard solution calibrants and AuNP suspensions contained 50 µg L^−1^ of thallium as internal standard. These solutions/suspensions were analysed by conventional ICP-MS using a Perkin Elmer Nexion 300D instrument (dwell time: 50 ms, sweeps per reading: 20). Three replicates were prepared for each AuNP material.

#### 2.5.2. Determination of Mass Concentration of Digested AuNP Suspensions

Three different laboratories determined the Au mass concentration after digestion with aqua regia. Two observed the following protocol: a calibration curve from ionic Au standard solutions was prepared in 1.5% nitric acid (blank, 5, 10, 15, 20 µg L^−1^). All solutions were prepared gravimetrically, using an intermediate solution of 10 mg L^−1^ ionic gold. The intermediate solution was obtained by applying a dilution of 1:100 of a 1000 mg L^−1^ Au standard solution. A total 100 µL of the sample suspensions (of an approximate Au mass concentration of 45–50 mg L^−1^) were weighed into 50 mL polypropylene test tubes. After addition of 0.15 mL concentrated nitric acid (65%) and 1.5 mL concentrated hydrochloric acid (37%), the suspensions were allowed to sit for one hour. After addition of thallium as internal standard (10 µg L^−1^), the digestion tubes were brought to volume (50 mL). This solution was then diluted 1:10 with 1.5% nitric acid before analysis. A blank sample underwent the exact same procedure, however without the addition of ionic Au standard solution. For quality control purposes, a recovery sample was prepared: 500 µL of 10 mg L^−1^ ionic Au standard solution was added to a 50 mL test tube. All following steps were the same as described earlier. A detailed protocol can be found in the Supplementary Materials (SM1). The third laboratory applied a slightly deviating protocol: 3 mL of concentrated hydrochloric acid (37%) and 1 mL of concentrated nitric acid (65%) were added to 10 µL of undiluted sample material. The suspensions were allowed to sit for two hours before addition of 15 mL ultrapure water. This solution was diluted five times before analysis with ICP-MS. All dilutions were done gravimetrically. While two laboratories performed the analysis using solely ICP-MS, one laboratory analysed samples with ICP-MS and ICP-OES. Details on the ICP-OES conditions are included in Supplementary Materials (SM2).

### 2.6. Determination of the Ionic Fraction of AuNP Test Materials

For the determination of the ionic fraction of the investigated AuNP suspensions, 50 µg L^−1^ samples were prepared in duplicate for each material. In addition, two blank samples (ultrapure water) and two recovery samples (60 µg L^−1^ ionic Au) were prepared. These suspensions/solutions were filtered (30 min at 4000 rpm) through Amicon-4 centrifugal filters (Merck Millipore, Amicon-4, Ultracel, 3K, Product code UFC800324) with filter membranes made of regenerated cellulose. Before use, the filters were first washed with ultrapure water then wetted with small volumes of the product to be centrifuged to remove the residual water. Au concentrations before and after filtration (in the filtrate) were determined against an external calibration curve (0–100 µg L^−1^ ionic Au standard solution) with ICP-MS operated in normal mode. Instrumental settings are described under Section 2.5.1.

### 2.7. Determination of Transport Efficiencies Based on TEF and TES Methods

Seven laboratories, listed in Table 3, determined transport efficiencies based on TEF and TES methods for the six AuNP suspensions included in this study following a specific protocol (SM3). The protocol was based on the indications provided in the technical specifications ISO/TS 19,590 [8].

AuNP working suspensions were gravimetrically prepared by dilution of the concentrated suspensions following the indicative schemes provided in Table S4a,b (SM4). The required dilutions were determined based on particle sizes and Au mass concentrations declared on the CoAs, or for those where this information was not available, by in-house measurements conducted in this study (Section 2.5). For the determination of TES, ionic Au standard solutions were prepared in 1.5 mM sodium citrate (blank, 1, 3, 5, 10 µg L^−1^). In the revised final protocol (SM3), it is suggested to prepare the ionic gold solutions in ultra-pure water, since ionic gold—after several hours—may reduce to elemental gold in the presence of citrate. The ionic response in water and in 1.5 mM proved to be identical (Figure S5, SM5). After analysis of both the ionic Au calibration standards and the diluted AuNP suspensions, TES was automatically displayed by the software based on the principles described in ISO/TS 19,590 [8]. From the same acquisition file, the particle flux in the plasma (number of particles detected per scan time) was derived. The corresponding particle number concentration reaching the plasma (particles mL^−1^) was then obtained by dividing the number of particles detected per scan time by the duration of the scan time [min] and the flow rate of the peristaltic pump [mL min^−1^]. Finally, TEF was manually derived by dividing the detected number of particles per mL reaching the plasma by the total amount of particles in the aspirated suspension, corresponding to the number of particles that would be detected if the transport efficiency was 100%. All samples were analysed in triplicate (three independent sample preparations) and on three different days. In some of the laboratories participating in the study, due to time constraints, some products were analysed in duplicate only.

#### Statistical Analysis

Size distributions were represented as histograms (bin size = 1 nm), absolute frequencies and cumulative distributions. Quantitative analysis by TEM and SEM was reported as means and medians of the minimum and maximum Feret diameter (Fmin, Fmax) and of the equivalent circular diameter (ECD). Uncertainties were expressed as expanded measurement uncertainty (coverage factor (k) = 2) at the 95% confidence level. Mass concentrations were reported as means and standard deviations. Normality of distributions for the correct selection of statistical tests was verified by the Shapiro–Wilk test [23]. The comparisons between AuNPs for each laboratory in terms of TES, TEF and the difference between TES and TEF were evaluated by the Kruskal–Wallis test, followed by the Bonferroni post hoc multiple comparison test [24]. The differences between TES and TEF were analysed by the Wilcoxon test. Transport efficiencies based on TES and TEF were illustrated by bar charts, grouped by laboratory and tested materials, or by tested materials and day of analysis. The correlations between transport efficiencies, evaluated for each AuNP, were measured by the Spearman rho correlation coefficient. The choice of the fitting model on the mean intensity used for the determination of transport efficiency, considered Gaussian, LogNormal and Max Intensity model fits, as available in the instrument’s software (Perkin Elmer/Syngistics). To evaluate the relationship between the particle concentration in the aspirated suspension and the number of detected particles/scan time, separately for N8151035 and AUCN60, regression models were run and the slopes were estimated. A *p* value <0.05 (two sides) was considered statistically significant. All statistical tests were performed by SPSS v.27 (IBM SPS Statistics software).

### 2.8. Determination of the Transport Efficiency (TES) with Particles Other Than Gold

TES can be determined with any element for which both well-characterised ionic and NP standards are available. Two pairs (ionic/particle suspensions) were selected for this study: platinum and silver. For comparison purposes, N8151035-AuNPs were additionally analysed in the same sequence. Specifications for these materials are included in Table 4.

The calibration range for ionic Ag and Pt standard solutions ranged from 1 µg L^−1^ to 10 µg L^−1^. The monitored isotopes and densities inserted in the software interface are detailed in Table 4. Suspensions were diluted to reach 100,000 particles mL^−1^. The NIST silver reference material (NIST RM 8017) was provided as lyophilised polyvinylpyrrolidone (PVP)-coated particle cake in a vial, which was reconstituted with 2 mL of ultrapure water before use. Following the instructions on the ‘Report of Investigation’ provided along with the reference material [25], the reconstituted suspension was not sonicated before use. The reconstituted silver concentration was nominally 1 mg mL^−1^. TES was determined applying various fitting curves to the obtained intensity frequency distributions. All solutions and suspensions were prepared in 1.5 mM sodium citrate. Except for those parameters included in Table 4, all other parameters were the same as described in Section 2.2.

### 2.9. Dilution Study with Multiple Levels

The dilution study was conducted with two AuNP test materials: N8151035 and AUCN60 (see Table 2). For each of these suspensions, dilutions were prepared gravimetrically to reach approximately 500, 1000, 2000, 4000 and 5000 particles per scan time. These dilutions were chosen as they fall within the range of applicability of the spICP-MS system used. All dilutions were prepared in 1.5 mM sodium citrate in duplicate. The number of detected particles per scan time (60 s) was compared against the expected particle number concentration in the aspirated suspension and the obtained transport efficiencies (TES and TEF). TES was computed as the ratio of RF_ionic_ to RF_NP_.

### 2.10. Determination of Transport Efficiency Based on the Dynamic Mass Flow Approach

The method suggested by Cuello-Nunez and co-workers [26] was reproduced on two ICP-MS instrument platforms, a Perkin Elmer Nexion 300D and an Agilent 8900. The Perkin Elmer instrument was equipped with a Meinhard concentric nebuliser and a baffled non-refrigerated cyclonic spray chamber. The Agilent instrument was equipped with a micromist concentric nebuliser and a Scott-type, double pass spray chamber cooled to 2 °C. Briefly, the DMF approach is performed by dynamically and continuously measuring the mass difference between the sample uptake and waste streams online over time whilst the ICP-MS system is in equilibrium, which is taken to represent the mass flow reaching the plasma. The transport efficiency value is then calculated as the ratio between the slope from the regression analysis representing mass flow reaching plasma (slope 1) and the slope from the regression analysis representing mass flow of sample uptake (slope 2). In practice, a vial with ultrapure water was placed on top of an analytical balance (4 decimal places) positioned close to the ICP-MS instrument. The sample uptake tube and the waste tube were both simultaneously placed in the aforementioned vial and left to stabilise for 30 min while the pump was running. Once stabilised, the weight of the vial was monitored every 3 min over a period of 45 min. In a second step, the waste tubing was removed from the vial. After stabilisation time of 15 min, the weight of the vial was recorded every 3 min over a total monitoring time of 15 min. The procedure was performed twice: once in the morning and once in the afternoon (Perkin Elmer Nexion 300D) or once on two different days (Agilent 8900). For comparison purposes, TES and TEF were also determined using N8151035 AuNPs.

## 3. Results and Discussion

### 3.1. Qualitative Analysis of AuNP Materials with spICP-MS

**Figure 1:**
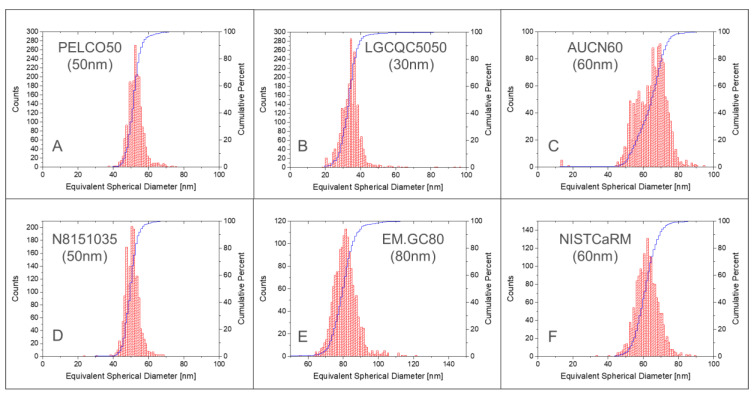
Number-weighted size distribution of gold particles in test samples (**A**–**F**) determined with spICP-MS. Histograms of absolute frequency (counts) and cumulative functions. Bin size: 1 nm.

Figure 1 depicts the number-weighted size distributions of all six materials used in this study. It appears that the product codes PELCO50 (Figure 1A) and N8151035 (Figure 1D) look very similar in terms of particle size distribution.

Both CoAs had indeed the same design although branded Ted Pella and nanoComposix (for the material distributed through Perkin Elmer). It seems probable that both are originally synthetised by nanoComposix and are then distributed by two different dealers. The two products differed primarily in their mass concentration. Product D had a much lower concentration (12.4 ng mL^−1^) and indeed was colourless, while all other suspensions showed the expected typical reddish colouration. An advantage of this lower-concentrated product is that it requires fewer dilution steps to reach the targeted particle number concentration for optimal spICP-MS analysis. This can be of interest to reduce possible errors related to the dilution steps when determining transport efficiency based on measured particle frequency. Compared to the other products, these two products show the narrowest size distribution. Products B, E and F show comparable broadness in size distribution, while product C has the broadest distribution and seem to contain two distinct sub-populations (around 55 nm and around 70 nm), despite the claim by the supplier that the suspension was monodisperse. Except this last material, all other products show monodisperse, Gaussian-like distributions of the particles.

### 3.2. Verification of Particles’ Size with Electron Microscopy

The particle size distribution of the six test materials was verified with both transmission electron microscopy (TEM) and scanning electron microscopy (SEM).

#### 3.2.1. Qualitative Analysis

**Figure 2:**
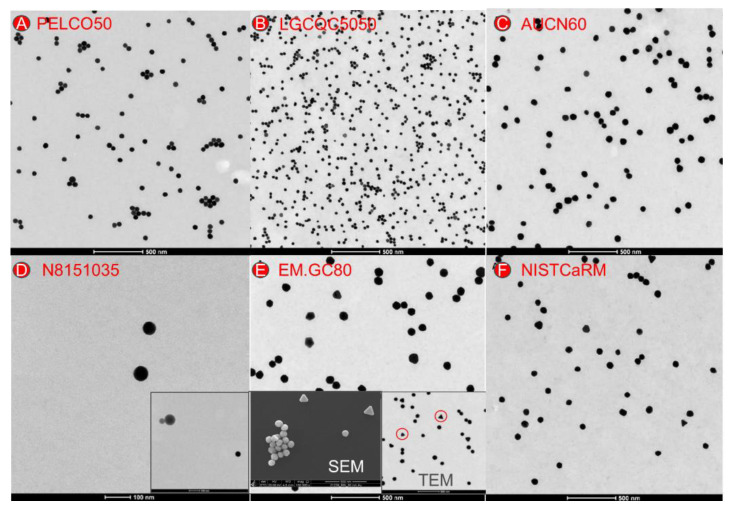
Annotated TEM/SEM micrographs resulting from the image analysis of the constituent particles of the six test materials (**A**–**F**). The insert for test material (**D**) shows-besides the gold particles-also some larger (80–100 nm) particles of lower density. Inserts for test material (**E**) show a subpopulation of triangular particles.

For all samples, the quality of the specimen preparation could be assessed, and the material present on the grid could be described in detail, based on representative electron micrographs (Figure 2). The equivalent SEM images can be found in SM6. TEM/SEM analysis revealed that test material EM.GC80 included a subpopulation of triangular particles (Figure 2E) which may result in a slight bimodal particle distribution. The strongly diluted material distributed by Perkin Elmer (N8151035) besides the gold particles, contained also some larger (80–100 nm) particles of lower density (Figure 2D, inserted image). Energy-dispersive X-ray analysis (EDX) of these unexpected particles in product N8151035 showed a strong sulphur signal (SM7), while at the same time they did not show the gold signal. The origin of these additional particles remained unclear.

#### 3.2.2. Quantitative Analysis

Graphs showing the constituent particle size distribution of the minimum Feret diameter, the (maximum) Feret diameter, the equivalent circular diameter and the aspect ratio are displayed in SM8. The statistics of the sample measurements are presented in Table 5.

Although in most cases CoAs do report a size value and its related coefficient of variation, they rarely specify the type of diameter that was determined (Fmin, Fmax, ECD) and rarely detail whether the indicated value corresponds to the mean-, the modal- or the median size.

Considering measurement uncertainties, the values determined in this study with TEM and SEM matched well with those declared on the CoAs. No correction for size was therefore deemed necessary for the determinations of TES and TEF.

### 3.3. Impact of Dispersant on Stability of AuNP Suspension

**Figure 3:**
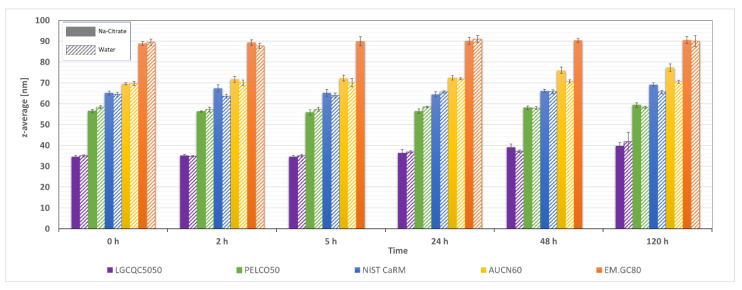
Assessment of suspensions stability. Measurement of z-average (DLS) at various time intervals after dilution of AuNP suspensions with ultrapure water or 1.5 mM sodium citrate.

The aim of these measurements was to assess whether diluting concentrated AuNP suspensions in either ultrapure water or in 1.5 mM sodium citrate has an effect on the stability of the suspension. Dilution can destabilise suspensions due to NP adsorption phenomena on vials or pipette tip surfaces [11]. The stability of the suspensions was assessed with DLS and with spICP-MS. For the DLS measurements, the particle concentration was chosen to be in the range of operability of DLS for the given particle sizes. For particles in a diameter range of 30–80 nm, concentrations of around 3 mg L^−1^ were found to be suitable. Results represented in Figure 3 show no relevant change of the hydrodynamic particle diameter (z-average) in the first 24 h, neither when dispersed in ultrapure water nor in 1.5 mM sodium citrate. At durations exceeding 24 h, particles from the LGCQC5050 and the AUCN60 test materials showed an increase of the hydrodynamic diameter corresponding to 5 nm and 8 nm respectively. Considering that the transport efficiency is among the first parameters determined on a day of spICP-MS measurements, results obtained by DLS indicate that diluting the samples either in ultrapure water or in 1.5 mM sodium citrate, does not have an impact on the stability of the suspension and therefore ultimately on the determination of the transport efficiency.

The concentrations required for DLS measurements were several orders of magnitudes higher than those typically required for spICP-MS analysis. Since this might have an effect on the interactions between the particles [27,28,29] and therefore eventually on the stability of the suspension, it was decided to assess the stability of the particle suspensions also at concentrations typically used for spICP-MS analysis. To this end, diluted suspensions of two of the test materials (AUCN60 and PELCO50) were prepared both in ultrapure water and in 1.5 mM sodium citrate. These suspensions were analysed by spICP-MS at various time intervals (1, 3, 5 and 8 h) and the cumulative number size distribution determined.

**Figure 4:**
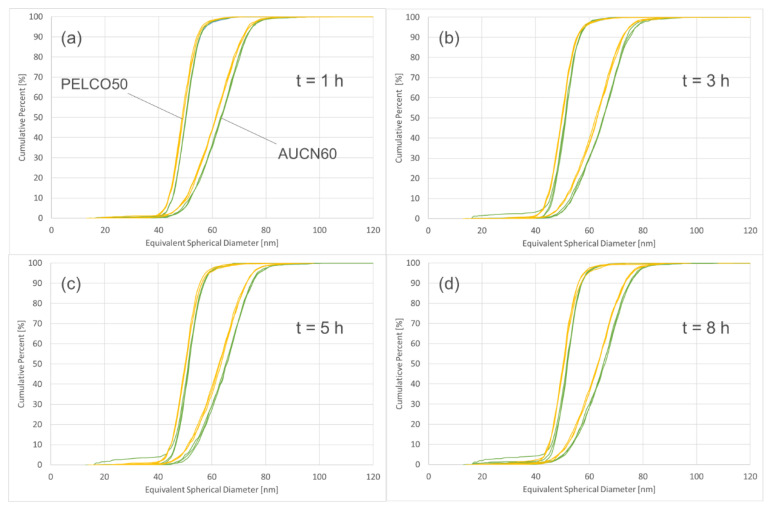
Cumulative number size distributions of surface treated and non-surface treated particles at various time intervals ((**a**) = 1 h, (**b**) = 3 h, (**c**) = 5 h, (**d**) = 8 h), diluted in ultrapure water (green lines) and in 1.5 mM sodium citrate (yellow line).

Results show that the particle sizes of samples prepared in sodium citrate are slightly smaller compared to those prepared in ultrapure water (Figure 4). At 50 percentile this difference corresponds to less than 1 nm for the PEGylated particles, while for the bare particles the difference corresponds to approximately 2–3 nm for all time intervals. Especially for the surface treated particles, the differences in size are so low that they can be neglected and play only a minor role in the overall uncertainty of a measurement obtained with spICP-MS. In the present study it was decided to proceed with preparing all particle suspensions in 1.5 mM sodium citrate.

### 3.4. Determination of the Mass Concentration of Digested and Non-Digested Test Material AuNPs Suspensions

Reliable knowledge of the gold mass concentration of the assessed products is essential to derive the accurate particle number concentration, which is crucial for the determination of TEF. In the frequency method, the theoretical particle concentration is derived from the mass concentration and the particle size. For all the test materials, except EM.GC80 and NISTCaRM, mass concentration values were provided on the CoAs.

Au mass concentrations of the products used in this study were determined both with and without prior digestion with aqua regia. The aim of this two-fold determination was to identify possible differences attributable to incomplete atomisation and ionisation of the particles in the plasma. The determination of the Au mass concentration following digestion revealed significant challenges. During the analysis of the blank sample, which was treated in the same way as the samples and therefore contained small residues of aqua regia, a strong increase of the baseline signal was observed. Long washing periods (>10 min) were necessary to reach acceptable baseline levels. Au is one of the elements suffering from memory effects associated with interactions with the sample introduction system [30,31,32]. Aqua regia or its reaction products seem to dissolve residues of elemental Au deposited in the sample introduction system (tubing walls, spray chamber, nebuliser) which are not released by aspiration of 2% nitric acid, i.e., the conditioning solution that is typically used. Recovery measurements were fundamental as quality control to avoid overestimation. One laboratory determined mass concentrations also with ICP-OES, which in some cases is less susceptible to small variations of the blank level related to gold washout from the introduction system. Table 6 includes all Au mass concentrations determined by the three laboratories with various techniques, with and without digestion. Considering the uncertainties of the obtained mass concentrations, for most of the tested materials the difference compared to the declared value is either irrelevant or very small. The product that showed the highest variability and deviation from the declared value was PELCO50. The declared value seemed to be lower compared to the average concentration of all three laboratories (53.0 mg L^−1^ vs. measured average of 55.2 mg L^−1^). For most of the tested products, the CoA did not include an uncertainty of the declared mass concentration, which would also need to be taken into consideration. Au concentration of NIST CaRM, for which no Au concentration value was provided, was found to be 51.3 mg L^−1^ on average. The second product for which no (direct) gold mass concentration was provided on the certificate was the BBI test material. In this study its concentration was determined as being 46.4 mg L^−1^ on average.

### 3.5. Determination of the Ionic Fraction

The presence of ionic Au in the suspensions used for the determination of transport efficiencies is undesired. This is especially true when the determination is based on measured particle frequency, where the theoretical number of particles is derived from the particle diameter and the suspension’s mass concentration. Thus, all AuNP suspensions were tested for the presence of a soluble gold fraction. No ionic Au could be detected in the filtrate for any of the materials. For quality control and recovery purposes, a 60 µg L^−1^ ionic Au standard solution (diluted in ultrapure water) was filtered as well. The recovery, measured on two separate days, was found to be 61.9 ± 0.9% (*n* = 2) and 66.8 ± 4.4% (*n* = 4) on the first and the second day respectively. Some of the ionic Au got lost during the filtration process, possibly due to adsorption on the filter membrane made of regenerated cellulose. Given that the recovery on the two investigated days was relatively repeatable and therefore assuming an average recovery of around 65%, a theoretical maximum possible amount of ionic Au in the starting products could be estimated from the limit of quantification of ionic Au. The limit of quantification was estimated as being equivalent to the concentration value that corresponds to an instrument signal-to-noise ratio in the range of 10 [33]. The noise corresponded to 73.2 ± 6.1 cps (*n* = 7). Ten times the noise was therefore equivalent to around 730 cps, which corresponded to an Au mass concentration of 0.05 µg L^−1^. Therefore, the measured Au concentrations in the filtrate of all tested products corresponded to <0.05 µg L^−1^, becoming 0.08 µg L^−1^ when adjusting for the average recovery. The analysed samples were diluted 1:1000. In the undiluted sample, the theoretical highest amount of ionic Au is therefore below 0.08 mg L^−1^ and can therefore be deemed to be irrelevant. An additional aspect that made it unlikely to find ionic Au in the test materials, is that Au ions such as tetrachloroaurate are reduced to elemental Au in the presence of citrate ions in aqueous solutions [34]. Most of the tested products were sold suspended in citrate solutions.

### 3.6. Determination of Transport Efficiencies Based on the TES and TEF Methods for Six AuNP Suspensions in Seven Expert Labs

**Figure 5:**
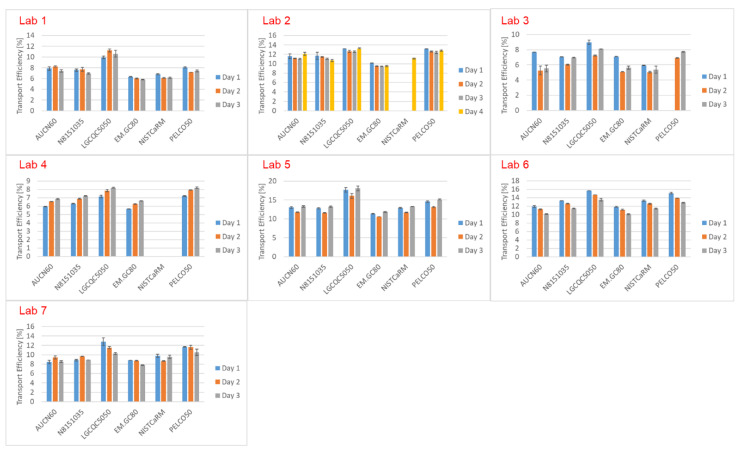
Transport efficiencies based on measured particle size (TES) determined on different days.

Figures 5, 7 and 8 summarise the transport efficiencies obtained by each of the participating laboratories based on measured particle size (TES) and particle frequency (TEF) for each measurement day. Values were obtained using the size and mass concentration values provided on the CoAs without applying any correction. Figure 5 depicts the TES results. While for some of the laboratories, TES obtained on different days of measurement matched well, even with more than one month between the first and the last day of determinations (Lab 2), other laboratories found wider variabilities. For some of the laboratories some trends were observed. Laboratory 4, for example, found systematically increasing transport efficiencies from the first to the third day, while laboratory 6 observed the opposite. A possible reason for this could be attributed to the tuning procedure of the ICP-MS, which between one day and the other could result in small variations in transport efficiency.

Another trend that can be observed is that the obtained transport efficiencies do generally match well independently of which AuNP was used, except when using product LGCQC5050. Here the transport efficiencies were systematically higher for all laboratories by 0.17% to 6.05% (average values for all laboratories and all measurements). More detailed statistical analysis is summarised in Table S9 (SM9).

**Figure 6:**
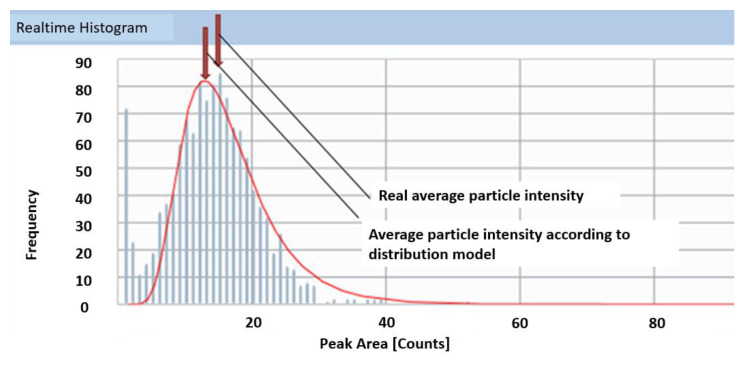
Nanoparticle intensity-frequency distribution histogram with fitting curve for product LGCQC5050.

An overestimation of transport efficiency by TES could be caused by an overestimation of particle size (mass) or an underestimation of the peak intensity of the reference particle. As the verification measurements conducted in this study confirmed the declared particle size, the explanation might be found in the setting of the average intensity of the reference particle. For the determination of the transport efficiency based on measured particle size, both ionic standards and particle suspensions of the same element (in this case gold) are measured. The transport efficiency is then obtained by dividing the ICP-MS response for ions (cps µg^−1^) and the response for nanoparticles (cps µg^−1^). The nanoparticle response in turn is defined by the mean reference particle intensity measured for the gold nanoparticles in the working standard divided by the mass of the nanoparticle. The mass of the nanoparticle is obtained from its size assuming perfect sphericity. The mean intensity of the reference particle is the intensity (ms dwell time analysis) or peak area (µs dwell time analysis with peak integration) that corresponds to the mass of the reference particle. The value is typically obtained based on a histogram of intensities or peak areas. Often a fit is applied to determine the most common intensity/peak area in the histogram (Figure 6). Test material LGCQC5050 contained particles with an average diameter of around 30 nm and the lower size-bound of the particle distribution close to the gold’s particle size detection limit. The smallest nanoparticle that can be detected using spICP-MS is determined by the sensitivity of the ICP-MS system and the ability to differentiate particle signals from the background signal. It corresponds to the point (threshold) where the extrapolated particles’ signal intensity equals the background plus three (or in some cases five) times the standard deviation [35]. In some cases, software interfaces do apply ‘patented algorithms’ for automated threshold detection. The closeness to the size detection limit sometimes results in a non-optimal fitting curve and hence in a shift of the average nanoparticle intensity used by the software to calculate transport efficiency towards lower values (Figure 6). It should however be noted that product LGCQC5050 is primarily supposed to be used for the TEF method as it is assessed for the particle number concentration.

**Figure 7:**
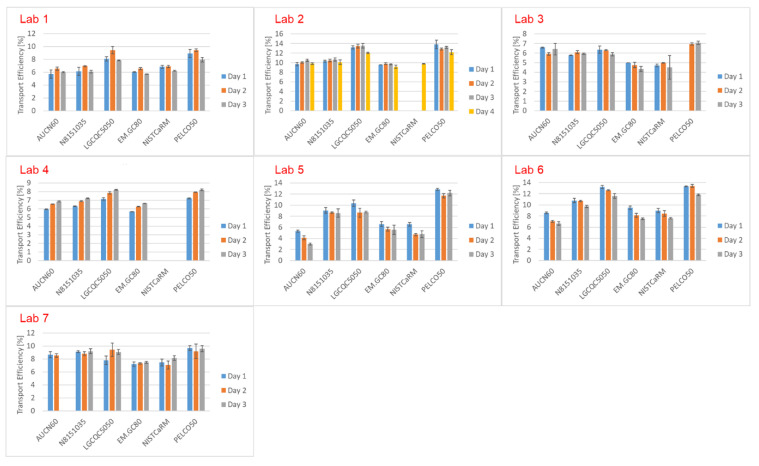
Transport efficiencies based on measured particle frequency (TEF) determined on different days.

Transport efficiencies determined on different days, based on measured particle frequency (TEF), are displayed in Figure 7. Transport efficiencies determined with products LGCQC5050 and PELCO50 were found to be systematically higher in most laboratories, compared to values obtained with the other test products (Table S9, SM9). For test product PELCO50, this could be explained by its effective mass concentration, which turned out to be higher compared to that indicated on the certificate of analysis (55.2 mg L^−1^ vs. 53.0 mg L^−1^ declared). Test product LGCQC5050, compared to all other products, contained the smallest particles. A small variation from the mass concentration declared on the certificate of analysis did therefore have a stronger impact on the theoretical number of particles derived from the mass concentration and the size. The certificate of analysis of product LGCQC5050 reports an assessed value for particle number concentration (1.47 ± 0.28 × 10^11^ kg^−1^) with a 95% confidence level [36], determined by spICP-MS. Since the transport efficiency was determined following the dynamic mass flow approach based on gravimetric determination of the NP mass flow, this value is traceable to the SI. The reported particle number concentration was approximately 13% higher compared to the derived value used in this study. This might also have contributed to overestimating TES. Due to the relatively high uncertainty of the particle number concentration reported on the CoA (around 20%) it was preferred to calculate using the derived value obtained in this study, derived through the mass concentration and particle size. Compared to the other laboratories, laboratories 5 and 6 found higher variations (up to 400%) among the transport efficiencies determined with each of the tested products. A possible reason is the degradation of the test materials during shipment. Particles may have adsorbed on the walls of the vials in which they were shipped. Stability tests were conducted in the same vessels over a time of three weeks in which no relevant degradation was observed (SM10). These stability tests were, however, conducted under stable conditions at room temperature which do not necessarily reflect the conditions to which samples were exposed during shipment. Adding temperature trackers to the parcels would have helped clarify this point. An important conclusion that can be drawn from these results is that transport efficiencies based on measured particle size proved to be more robust as TES is not (or is less) affected by particle losses due to adsorption phenomena.

**Figure 8:**
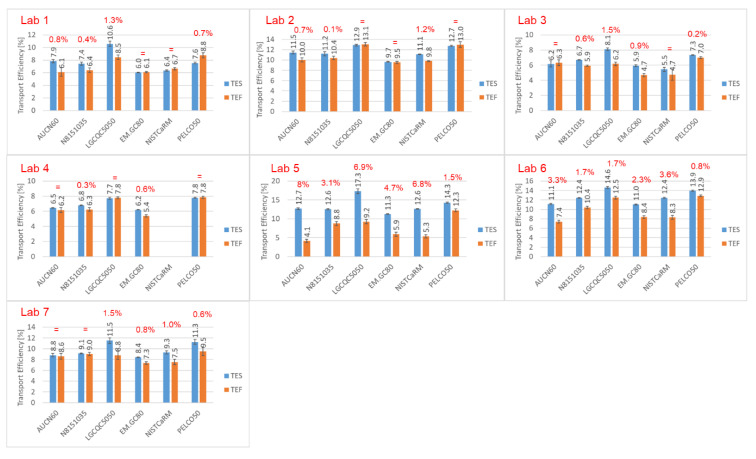
Transport efficiencies obtained based on measured particle frequency and particle size as an average of all three/four days of measurement. The red percentages at the top of the bars represent the absolute differences between the two approaches considering the lower and upper uncertainty bounds. Significant correlations between TES and TEF are shown in Figure S11 (SM11).

Figure 8 compares absolute differences between TES and TEF methods as an average of all three/four days of measurements. A general observation is that, if deviating, TES (blue bars) was always higher than TEF (orange bars) and, according to the experience of the different labs involved, TES values were closer to their usual TE values. As mentioned earlier, this may be attributed to the lower susceptibility of TES to sample degradation such as adsorption phenomena.

Considering also the upper and lower uncertainty bounds, it appears that for five out of the seven laboratories, no significant differences between TES and TEF were observed. Laboratories 5 and 6 found much higher variations, reaching TES/TEF ratios of up to 300% (*p* < 0.01, Table S9, SM9). Longer shipping times and prolonged non-controlled storage conditions of the test materials might have been the cause of these differences.

### 3.7. In-Depth Investigations

Based on the outcomes of the interlaboratory study (Section 3.6), a number of in-depth investigations were conducted in only one or two of the laboratories.

#### 3.7.1. Correction of Transport Efficiencies by Mass Concentration and by Size

One of the objectives of this study was to verify both the mass concentrations and the particle sizes declared on the CoA. This is relevant as inaccurate values have an impact on the correct determination of transport efficiencies. The outcomes of these verifications (Section 3.2.2 and Section 3.4) showed that the majority of values reported on the certificate of analysis matched well with those determined in this study. Only minor deviations were observed. These are summarised in Table 7.

Biases in the mass concentration affect solely the determination of TEF. Since derived from the declared mass concentration and the particle size, the calculated absolute number of particles in the aspirated suspension would be inaccurate as would the determined transport efficiency. On the other hand, deviations from the declared particle size would affect both TEF and TES. It is important to note that a bias in the mean particle size would impact the determination of both TEF and TES by the power of 3, while inaccuracy of the mass concentration would only have a linear impact on TEF.

**Figure 9:**
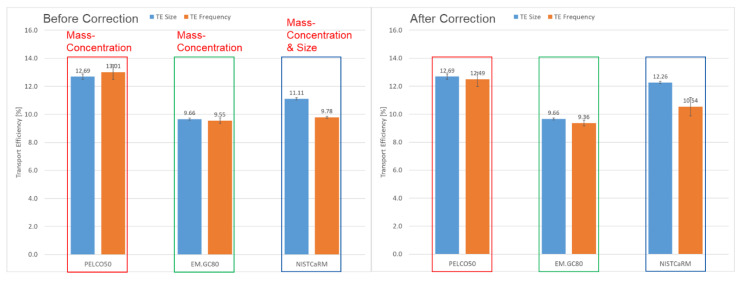
Transport efficiencies before and after correction for mass concentration and particle size.

Figure 9 shows on the left side transport efficiencies determined without applying any correction for mass concentration and size, using the values provided on the CoA, and on the right side, transport efficiencies with corrected values. This simulation was completed using the data from laboratory 2 only. Test materials PELCO50 and EM.GC80 were corrected for mass concentration only. In both cases the experimentally determined mass concentrations were higher compared to those indicated on the certificate and this resulted in corrected transport efficiencies (TEF) being slightly lower. In the case of the PELCO50 test material, this brought the values for TES and TEF even closer. For test material EM.GC80, the difference in mass concentration was very low and therefore only marginally affected transport efficiency.

For the NIST test material no reference values were provided. Only nominal values for mass concentration (around 50 mg L^−1^) and size (around 60 nm) were suggested. More precise values were obtained during the verification measurements. For this material, the correction was done for both the mass concentration and size. The re-calculated TEF increased by approximately 0.75% (absolute difference). The recalculation of the corrected TES resulted in an increase of approximately 1.15% (absolute value). For the NIST material the difference between TES and TEF therefore persisted also after correction for size and mass concentration.

#### 3.7.2. Impact of Fitting Curve on Transport Efficiency (Size Approach)

**Figure 10:**
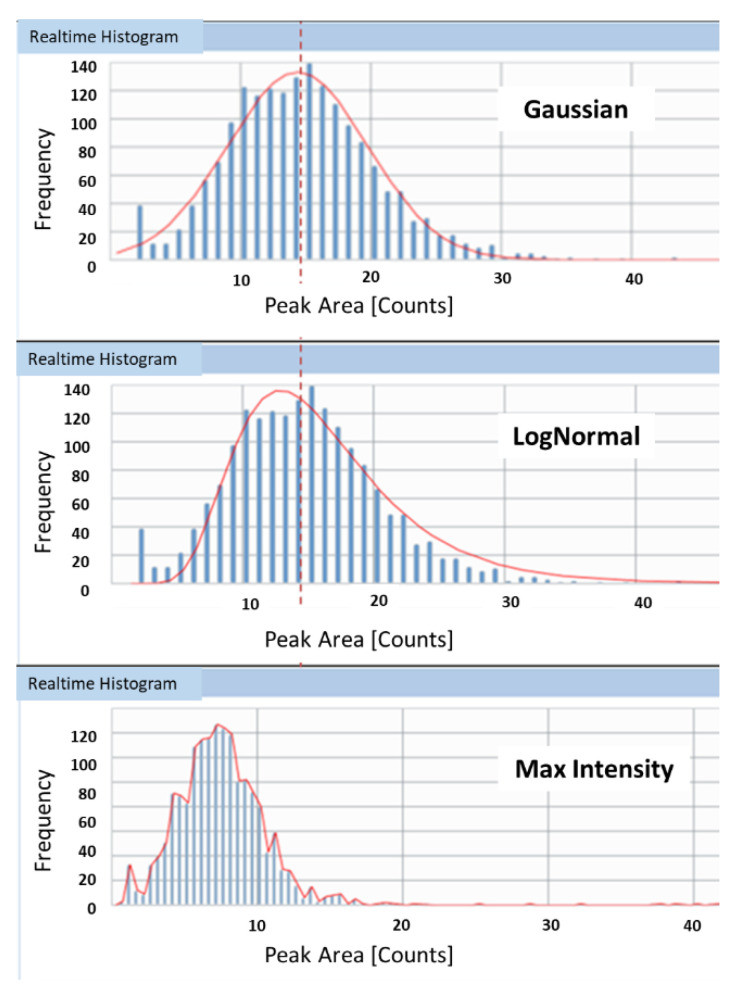
Impact of the choice of fitting model (red non-dashed line) on the mean intensity used for the determination of transport efficiency.

For the determination of TES, ionic standard solutions and NP suspensions of the same element are measured and their respective responses divided with each other. Since not all particles have exactly the same size, during the aspiration/measurement of the diluted nanoparticle suspension, the particles’ peak area (PA) counts are plotted against their frequency (Figure 10). The nanoparticle response is then obtained using the mean nanoparticle intensity of the distribution, which in turn—for the instrument/software (Perkin Elmer/Syngistix) used for this study—is derived from a model’s fitting curve. Depending on the model that is applied to the distribution, the value of the mean intensity—used by the software to calculate the transport efficiency—can slightly vary. It is important to note that the peak area/frequency histogram is a function of the peak area (PA) and not of the equivalent spherical diameter (ESD). The ESD is not linearly proportional to the peak area, but proportional to the cubic root. No matter how narrow and Gaussian the distribution of the nanoparticle standard is, there will always be a small difference in the determination of transport efficiency depending on the type of fitting model chosen.

**Figure 11:**
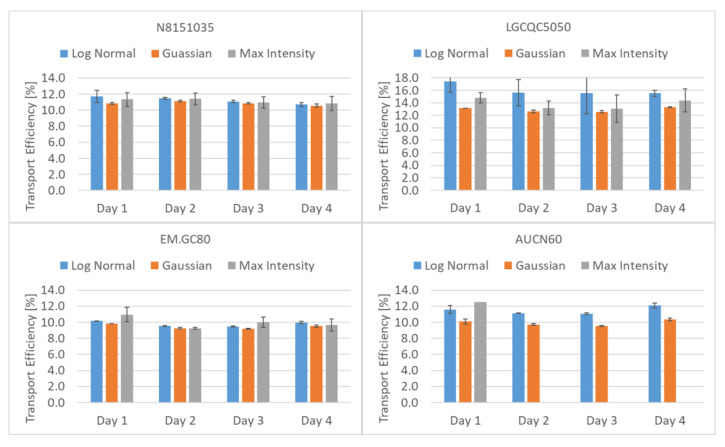
Transport efficiencies obtained (size approach, TES) by applying different fitting models. The software interface was unable to determine values applying the “Max Intensity method” for product AUCN60 on days 2–4.

However, nanoparticle standards with a narrow size distribution produce Gaussian-like frequency distributions as a function of the peak area. In this case, the Gaussian model and the Log Normal model provide similar results for transport efficiency. In practical terms, the less narrow and Gaussian the size distribution of the measured standard material is, the less the Gaussian model also fits. In those cases, the best of the available fitting model should be used. Figure 10 shows an example for test material LGCQC5050. In the upper part of the figure a Gaussian and in the central part a LogNormal model were applied. It can be observed that mean intensities do shift slightly. Some software packages also allow applying a ‘Max Intensity’ model, which in most cases is irrelevant. Figure 11 shows the impact the choice of the fitting model has on the determination of transport efficiencies based on measured particle size. This is shown for four of the tested materials, as averages of three replicates. As discussed above, for materials with a narrow and Gaussian-like particle number distribution (N8151035 and EM.GC80), the differences in obtained transport efficiencies is negligible. For less monodisperse materials such as AUCN60, the choice of fitting model does have an impact on the value ultimately determined. As discussed in Section 3.6, also for a material such as LGCQC5050, with a lower distribution bound close to the size detection limit (background), the choice of fitting model requires expert evaluation.

Not all instruments are accompanied by software interfaces that allow for choosing fitting curves. In other instruments/software packages the lower and upper limit of the data range is chosen and then the frequency weighted average peak area for this range is used instead.

#### 3.7.3. Determination of Transport Efficiency with Particles of Different Chemical Composition

**Figure 12:**
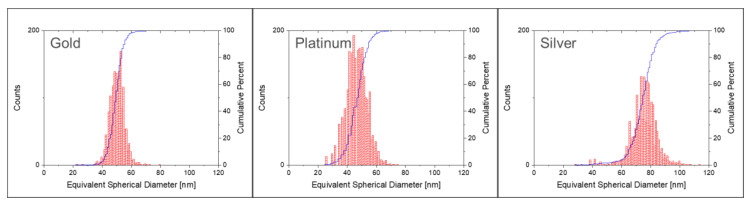
Number size and cumulative distributions (by number) determined with spICP-MS.

In principle, TES can be determined with any element for which both well characterised NP and ionic standard solutions are available. Whereas ionic standard solutions for ICP-MS exist for most elements, equivalent NP suspensions of the same element are scarce. In this set of measurements, TES was determined with suspensions of AgNPs and PtNPs. For comparison purposes, N8151035 AuNPs (Table 2) were analysed in the same sequence. The aim of this set of measurements was to investigate the possible effect of the chosen type of particle on the determination of transport efficiency. Figure 12 shows the number size distributions of the three tested materials, determined with spICP-MS. All three materials showed relative monodispersed size distributions. Silver and platinum NPs were slightly more broadly distributed.

For all materials, three replicate measurements were done. TES obtained with gold and silver were comparable, with a difference of approximately 1%, also taking the measurement uncertainties into account (Table 8).

While the good matching of transport efficiencies for N8151035 AuNPs and NIST RM 8017 indicates the equal suitability of both, significant differences were obtained with PtNPs, that resulted in TES being approximately 4.5–5% higher (absolute percentage).

This discrepancy can be explained by the structure of the PtNPs. The electron microscopy images provided on the certificate of analysis [37] shows that the PtNPs are porous aggregated structures, rather than hard spheres. This has also been observed in studies conducted in the past [38,39]. Since PtNPs are not solid, the particle mass is overestimated. The average nanoparticle intensity obtained during the measurements does, in fact, correspond to a smaller equivalent solid sphere. Consequently, the ICP-MS response to PtNPs is overall biased low resulting in TES biased high. A better characterisation of the porosity and a rigorous determination of the particle mass using alternative analytical techniques would be required before these PtNPs can be used as calibration standards for reliable determination of TES.

#### 3.7.4. Dilution Study with Multiple Levels

**Figure 13:**
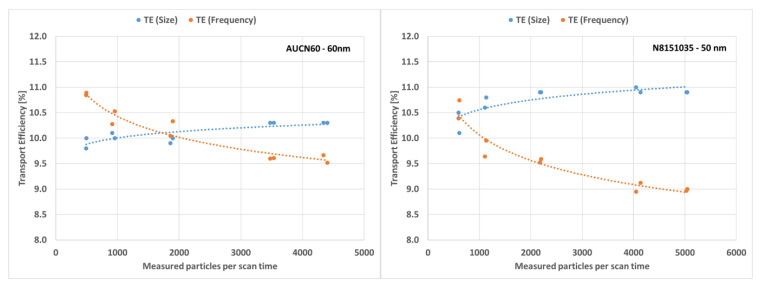
Impact of measured particles per scan time and calculated transport efficiencies based on frequency and size.

The purpose of this dilution study was to assess the impact of various dilution levels on the recovery of particles and ultimately on the determination of transport efficiencies. Measurements were completed with two of the test materials: N8151035 and AUCN60. These were chosen as they represent the two extremes in terms of particle distribution broadness among the tested materials. Figure 13 depicts the transport efficiencies (TES and TEF) calculated for each sample and replicate. It appears that for both sample materials TES is more reproducible over the entire dilution range. It ranged from 10.1% to 11.0% (diff. 0.9%) and from 9.8% to 10.3% (diff. 0.5%) for the N8151035 and the AUCN60 materials respectively. TEF ranged from 8.9% to 10.7% (diff. 1.8%) and from 9.5% to 10.9% (diff. 1.4%) for the N8151035 and the AUCN60 materials respectively. TES stabilised at more than 1000 particles per scan time. Below this number of detected particles, the impact of measurement uncertainty started to become relevant. An indication for this is the relatively large difference between the two replicate measurements. TEF shows a decreasing trend with an increasing number of particles per scan time, possibly due to the increasing probability of multiple particle detection events.

**Figure 14:**
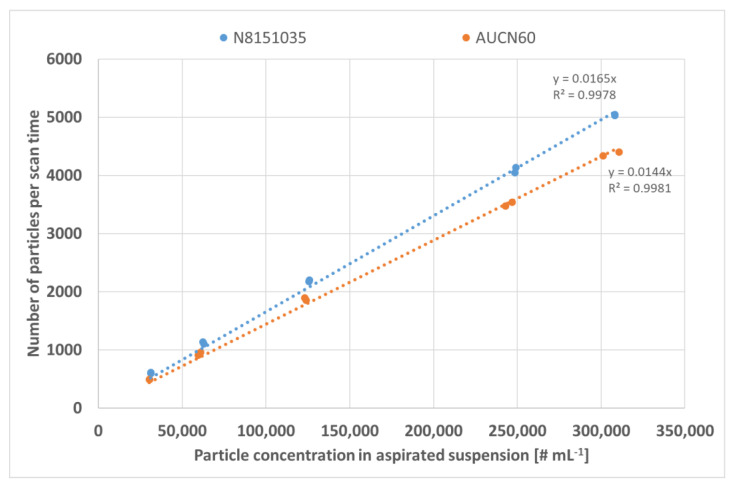
Relation between the theoretical calculated number of particles in the analysed suspensions and the detected number of particles per scan time.

By plotting the particle concentration in the aspirated suspension against the number of detected particles/scan time, which is nothing more than the definition of TEF, this effect becomes less evident (Figure 14).

Another effect that can be observed in Figure 14 is the non-matching of the slopes for the two investigated materials. This might indicate that the theoretical particle concentration calculated for each dilution is incorrect for either one or both of the products. This was, however, not confirmed by the results in Section 3.2.2 and Section 3.4. As described in Section 3.2.1, test material AUCN60 shows a vaguely bi-modal particle/size distribution. The conversion from mass concentration and size to number concentration might therefore be more biased for this material. In conclusion, dilution seems to have only a minor impact on the determination of transport efficiency as long as the number of detected particles/scan time are in the operational range of the instrument.

#### 3.7.5. Alternative Determination of Transport Efficiency by the DMF Approach

In this study, we replicated the DMF method and obtained transport efficiencies of 22.3% and 24.6% for two measurements on the same day with the Perkin Elmer Nexion 300D instrument (SM12) equipped with the cyclonic spray chamber, operated at ambient temperature. For comparison purposes, TEF and TES were determined using N8151035 AuNPs test material as a sample. Those approaches resulted in transport efficiencies of 10.7% for TES and 9.8% for TEF. When re-measuring the AuNP test material as a sample, mean sizes of 50 nm, 49 nm and 65 nm were obtained by the application of TES, TEF and transport efficiency determined by the DMF approach, respectively. This demonstrated that applying the DMF method resulted in an incorrect value for the transport efficiency with the Perkin Elmer Nexion 300D instrument under the given experimental conditions. With the Agilent 8900 instrument (Scott spray chamber operated at 2 °C), the transport efficiencies by the DMF method were 6.73% (day 1) and 7.42% (day 2). The corresponding TES and TEF using N8151035 were 6.4% and 5.8% on day 1 and 6.7% and 6.3% on day 2. Only with the Agilent 8900 instrument did the TES, TEF and DMF methods provide comparable values, while with the Perkin Elmer Nexion 300D instrument the DMF method provided twice the transport efficiency compared to TES and TEF. A possible explanation could be offered by the different configuration and temperatures of the two sample introduction systems. While the Perkin Elmer Nexion 300D was equipped with a non-cooled cyclonic chamber, the Agilent instrument was equipped with a cooled (2 °C) Scott-type spray chamber, very similar to the system used by Cuello and co-workers [26]. The DMF approach is an indirect method for the determination of the transport efficiency. It assumes that the entirety of the aspirated sample suspension leaves the sample introduction system through the plasma and the waste tube. It does not account for droplets deposited on the walls of the sample introduction system or a part of the sample evaporating via the argon gas [11,40]. Cooling of the inlet system (2 °C) may affect the performance of the spray chamber; it decreases the volatility of solvents and leads to faster washout. The advantage of the DMF-based over the reference material-based methods (TES and TEF) is that it does not require a NP standard or (certified) reference material. However, the accuracy of the DMF method has been documented to date only by using a conventional Micromist nebuliser with a cooled Scott-type spray chamber at 2 °C. The method has not been reported to work outside these conditions. Results of this study indicate that at room temperature and using a cyclonic chamber, the DMF is significantly biased high resulting in inaccurate particle number concentration and size determination for AuNP samples. A systematic study with different types of nebulisers, different types of spray chambers operated at different temperatures, injectors and different types of instrument platforms would help to better define the field of applicability of the DMF method.

### 3.8. Impact of Variations of Transport Efficiency on Particle Size and Number Concentration

**Figure 15:**
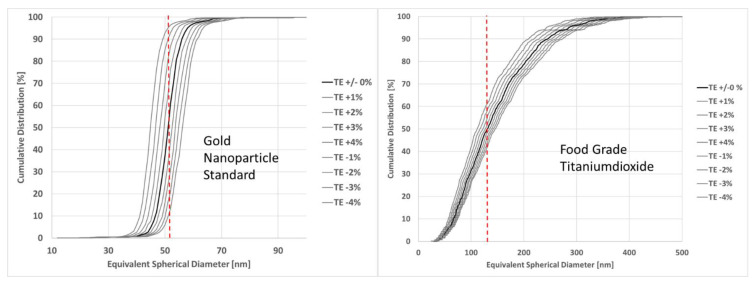
Variations of particle number size distributions as a function of increments/decrements of transport efficiency for a monodisperse and a polydisperse material. The red dashed lines represent the D50 value of the distributions with no TE deviation.

The previous sections discussed the impact of various parameters (bias on the mass concentration and particle size, choice of fitting curve) on the variation of transport efficiency and therefore ultimately also on the determination of particle size and particle number concentration of nanoparticulate samples. This section discusses the extent of the impact of inaccurate transport efficiency on sizing and number concentration determinations. To this end, a monodisperse gold (PELCO50) sample and a polydisperse food grade titanium dioxide sample used in past studies [41] were chosen for demonstration purposes. The thicker lines in Figure 15 represent the number size distribution of particles obtained with transport efficiencies determined for these two samples in past studies (TES using AuNPs) used as a reference. The lighter lines correspond to the size distributions with increments/decrements of transport efficiency in 1% steps (absolute values). One absolute per cent corresponds to approximately 8% and 9.5% in relative deviation for the gold and the titanium dioxide samples respectively.

Size distributions were obtained by manually changing the value of the transport efficiency directly in the software interface (Perkin Elmer, Syngistix v2.5) and then plotting each of the resulting distributions. The two samples were analysed on a Perkin Elmer NexIon 300D. Details of settings are described in Section 2.2. Results show that for the monodisperse material (Figure 15, left side) each 1% increase/decrease of transport efficiency resulted in an increase/decrease of the median diameter (D50) of 1 nm to 1.5 nm. For the polydisperse material (Figure 15, right side), the increase corresponded to 3–5 nm for each percentage of change.

Concerning the impact on particle number concentration, Table 9 shows the absolute and the relative variation with each percentage of increased/decreased transport efficiency.

It is noted that the variation of transport efficiency that can be deemed acceptable, depends on the measurement aim.

## 4. Conclusions

This study systematically investigated criteria that affect the determination of the transport efficiency, a parameter required for single particle ICP-MS analysis. Transport efficiency can be determined based on either the measured particle size (size approach) or the measured particle frequency (frequency approach). The two approaches should theoretically be equivalent; this study showed, however, that in practice this is not always the case. Although the difference between transport efficiencies obtained following the size and the frequency approaches under ideal conditions (e.g., no alteration of particle number or size) did not deviate much, use of the frequency approach (TEF) systematically resulted in lower transport efficiencies. The relevance of these differences does, however, depend on the purpose of the measurement.

Both the size and frequency methods rely on the accurate knowledge of the pump’s aspiration flow, the particle’s size, and both are based on the assumption that particles are solid spheres (i.e., non-porous).

The size method additionally requires the ionic solution and the particles to behave similarly during nebulisation, atomisation and ionisation. This is usually the case for particles with a diameter smaller than 100 nm. Moreover, this study demonstrated the relevance of using standard suspensions with Gaussian-like particle distributions that are as monodisperse and narrow as possible. The frequency method additionally relies on the accurate knowledge of the nanoparticle number concentration in the standard suspension, which is usually derived from the mass concentration, density and size of the particles. This study showed that in some cases the information provided on the certificates of analysis requires verification. In addition, inaccurate dilutions have a stronger impact when applying the frequency approach.

With awareness of these parameters, many of them can either be eliminated or controlled. An aspect that is difficult to control and that turned out to be relevant in this study is particle losses occurring on the way from the aspirated suspension until atomisation in the plasma. Particle adsorption on pipette tips, sample tubes and in the sample introduction system are hard to control.

As an overall conclusion, if the primary aim of spICP-MS analysis is sizing, transport efficiency should preferably be determined based on measured particle size, since it is not impacted by particle losses caused by adsorption phenomena. The prerequisite is, however, the availability of a standard material with a narrow and Gaussian distribution of well-characterised particles. On the contrary, if the main aim of the measurement is the determination of the particle number concentration, then the frequency approach could be preferred, as it might better account for particle losses in the sample introduction system. The dynamic mass flow approach, an alternative to TES and TEF, was assessed as well in this study. The accordance of transport efficiencies obtained by TES, TEF and DMF depended on the instrumental configuration. A systematic follow-up study with different instrumental configurations (nebulisers, spray chambers) would be necessary to better define the field of applicability of the DMF method.

## Figures and Tables

**Table 1:** Overview of factors that affect the determination of transport efficiency (η_n_) based on measured particle size/frequency and influences studied in this work.

Variable	Particle Size Method (TES)	Particle Frequency Method (TEF)
Parameters that need to be known for the reference particle in advance	Particle mass (m_p_), typically calculated from average particle size (d) and density (ρ), assuming that NPs are spherical and solid	Particle number concentration (C_p_), typically calculated from particle mass (m_p_)/particle size (d) and mass concentration (C_m_) of the reference particle suspension, assuming all analyte present in NP form
Parameters that need to be measured/ determined during the spICP-MS experiment	Sample flow rate (V) Average intensity for the reference particle (I_p_) (background intensity subtracted) Response factor (RF) defined as the slope of the calibration curve average intensity (I_ionc_) over ionic concentration (C_ionic_) for the corresponding ionic solution (RF_ionic_)	Sample flow rate (V) Particle flux in the plasma (q_p_)
Equation for determination of transport efficiency η_n_	η_n_ = [RF_ion_/(t_dwell_ × V)]/RF_NP_	η_n_ = q_p_/V × C_p_
Important factors/sources of bias studied in this work	Accuracy of particle size (d) Stability of particle suspension (→ size d) Size distribution of reference material Fitting model used to determine the average intensity of the reference particle	Accuracy of particle size (d) and mass concentration (C_m_) Stability of particle suspension (→ number concentration C_p_)

**Table 2:** Gold nanoparticle suspensions used in this study. In the first column, in bold, product identification names used in all tables and figures (TEM = transmission electron microscopy; PTA = particle tracking analysis).

Material [Manufacturer, Description, Product Code]	Declared Size [nm]	Declared Mass Concentration [µg mL^−1^]	Declared Number Concentration [Particles mL^−1^]	Particle Surface	Dispersant
nanoComposix Gold Nanospheres, Citrate, NanoXact Product Code **AUCN60**	60 ± 7 (TEM)	54	2.3 × 10^10^ (Calculated)	Bare (Citrate)	2 mM Sodium-Citrate
Gold Nanospheres, PEG-COOH Distributed through Perkin Elmer, synthetised by nanoComposix Product Code **N8151035**	49.6 ± 2.1 (TEM)	0.0124	9.89 × 10^6^ (Calculated)	PEG Carboxyl	Aqueous 1 mM Citrate
LGC Limited Colloidal gold nanoparticles Product Code **LGCQC5050**	32.7 ± 2.0 (PTA)	45.1 ± 1.5 [µg g^−1^]	1.47 × 10^11^ ± 2.8 × 10^10^ [particles g^−1^] (determined with spICP-MS. Traceable to SI) ^2^	Bare (Citrate)	Sodium Citrate
BBI Solutions Gold Colloid Product Code **EM.GC80**	78.8 ± 6.3	Not declared	Not declared	Citrate Capped	Suspended in water, no preservative
Ted Pella PELCO Gold Nanospheres, PEG Carboxyl, Highly Uniform, **PELCO50**	51 ± 2 (TEM)	53	3.9 × 10^10^ (Calculated)	PEG Carboxyl	2 mM Sodium Citrate
NIST candidate reference Material ^1^ **NISTCaRM**	Nominal diameter 60 nm	Not declared	Not declared	Citrate stabilised	Not declared

^1^ The material provided by NIST was a candidate RM, citrate stabilised AuNPs with nominal diameter of 60 nm. No detailed information on size and concentration was provided for this material. ^2^ Traceable to the SI through gravimetric determination of the sample mass flow, gravimetric preparation of nanoparticle sample dilutions and gravimetric determination of the nanoparticle TE.

**Table 3:** Participants, instruments, instrumental settings and software packages.

Name of Laboratory	Instrument	Software	Pump Flow Rate [mL min^−1^]	Dwell Time [µs]	Nebuliser and Spray Chamber
Istituto Superiore di Sanità—Rome/Italy	Perkin Elmer Nexion 350D	Syngistix V2.5	0.30–0.32	100	Meinhard concentric nebuliser, baffled glass cyclonic spray chamber
Joint Research Centre of the European Commission— Ispra/Italy	Perkin Elmer Nexion 300D	Syngistix V2.5	0.15–0.18	100	Meinhard concentric nebuliser, baffled glass cyclonic spray chamber
Max Rubner-Institut (MRI)—Karlsruhe/Germany	Thermo iCAP Q	Thermo Qtegra with npQuant plugin	0.32–0.34	3000 (Total acquisition time 120 s)	PFA-ST MicroFlow nebuliser, quartz cyclonic spray chamber cooled to 2 °C
National Food Institute, Technical University of Denmark	Agilent 8900	Single Nanoparticle Application Module of the Agilent ICP-MS MassHunter software 4.6	0.31–0.32	100	Micromist (borosilicate glass) concentric nebuliser, Scott type, double pass (quartz) spray chamber cooled to 2 °C
National Institute of Standards & Technology (NIST)—Gaithersburg/USA	Perkin Elmer 350D	Syngistix V1.1	0.14–0.17	100	Meinhard TR-50-C0.5 micro-concentric glass nebuliser, glass baffled cyclonic spray chamber cooled to 2 °C
Service Commun des Laboratoires (SCL)—France	Perkin Elmer 2000	Syngistix V2.5	0.23–0.27	100	Micromist nebuliser with 0.4 mL/min nominal liquid flow rate, baffled cyclonic spray chamber cooled to 5 °C
Wageningen Food Safety Research (WFSR)	Perkin Elmer 2000	Syngistix V2.5	0.101–0.106	100	MicroFlow type c PFA-ST3 nebuliser (low pressure), high sensitive SilQ cyclonic Spray chamber for 2000, cooled to 3 °C

**Table 4:** Specification of ionic/particle standards.

Element	Ionic Solution	Particle Suspension				
	Name, Product Code	Name, Product Code	Declared Diameter (TEM) [nm]	Declared Concentration	Monitored Isotope [m/z]	Assumed Density [g cm^−3^]
Silver	Sigma-Aldrich, Silver standard for ICP, TraceCERT, Product code 12818.	NIST Reference Material 8017	74.6 ± 3.8 nm	2.162 ± 0.020 mg in vial (reconstituted with 2 mL of ultrapure water)	107	10.49
Platinum	Sigma-Aldrich, Platinum standard for ICP, TraceCERT, Product code 38168.	nanoComposix, 50 nm Platinum Nanoparticles, Citrate, NanoXact, Product Number: PTCN50	46 ± 4 nm	52 µg mL^−1^	195	21.45

**Table 5:** Summary of the mean and median values of the minimum Feret diameter, the Feret diameter and the equivalent circular diameter obtained from the quantitative TEM and SEM analysis of the constituent gold particles of the tested materials.

	TEM				SEM				
Test material	Number of Analysed Particles	Minimum Feret Diameter [nm] ^1^	Maximum Feret Diameter [nm] ^1^	Equivalent Circular Diameter [nm] ^2^	Number of Analysed Particles	Minimum Feret Diameter [nm]	Maximum Feret Diameter [nm]	Equivalent Circular Diameter [nm]	Declared Size [nm] ^3^
**AUCN60** Mean Median	544	60 ± 7 62 ± 7	67 ± 7 68 ± 8	64 ± 7 65 ± 7	1046	56 ± 7 57.0	65 ± 7 66.4	63 ± 7 63.9	61 ± 7 (TEM)
**LGCQC5050** Mean Median	7535	31 ± 4 31 ± 4	35 ± 4 35 ± 4	33 ± 4 33 ± 4	288	34 ± 2.6 34.1	40 ± 4 40.3	39 ± 2 38.7	32.7 ± 2.0 ^4^ (PTA)
**PELCO50** Mean Median	1281	49 ± 6 49 ± 6	52 ± 6 52 ± 6	50 ± 6 50 ± 6	266	50 ± 3 49.7	56 ± 5 55.9	52 ± 3 52.9	51 ± 2 (TEM)
**N8151035** Mean Median	51	51 ± 6 50 ± 6	54 ± 6 53 ± 6	52 ± 6 51 ± 6	211	48 ± 3 47.4	51 ± 4 49.8	49 ± 3 49.3	49.6 ± 2.1 (TEM)
**EM.GC80** Mean Median	312	84 ± 10 83 ± 10	93 ± 11 91 ± 11	88 ± 10 87 ± 10	306	82 ± 39 80.7	93 ± 9 91.2	88 ± 38 86.3	78.8 ± 6 (not specified)
**NISTCaRM** Mean Median	518	62 ± 7 62 ± 7	70 ± 8 69 ± 8	66 ± 7 65 ± 7	362	59 ± 6 59	68 ± 11 67	66 ± 7 65	60 (nominal)

^1^ Values ± expanded measurement uncertainty (k = 2; 95%) Ucx = 12%; ^2^ Values ± expanded measurement uncertainty (k = 2; 95%) Ucx = 11%; ^3^ Declared size on certificate of analysis accompanying the tested product; ^4^ Modal diameter. Value obtained using particle tracking analysis (PTA).

**Table 6:** Gold mass concentration of assessed products (declared on certificate, determined without digestion and determined following digestion). Mass concentrations were determined by three different laboratories. All uncertainties correspond to the standard deviation.

		Laboratory A		Laboratory B		Laboratory C
Product	Declared Mass Concentration [mg L^−1^]	Mass Concentration Non-Digested [mg L^−1^]	Mass Concentration Digested [mg L^−1^]	Mass Concentration Digested [mg L^−1^]	Mass Concentration Digested [mg L^−1^]	Mass Concentration Digested [mg L^−1^]
Analytical Technique		ICP-MS	ICP-MS	ICP-MS	ICP-OES	ICP-MS
Average Au spike recovery		107% ± 3%	107% ± 3%	110% ± 5%	104% ± 3%	94% ± 1%
AUCN60	54	53.2 ± 0.9 (*n* = 3)	54.2 ± 0.3 (*n* = 3)	54.3 ± 0.3 (*n* = 2)	53.0 ± 0.5 (*n* = 2)	52.5 ± 0.6 (*n* = 3)
N8151035	12.4 µg L^−1^	12.7 ± 0.1 µg L^−1^ (*n* = 3)	n.d.	n.d.	n.d.	n.d.
LGCQC5050	45.1 ± 1.5	48.0 ± 1.0 (*n* = 3)	45.0 ± 0.3 (*n* = 3)	46.1 ± 0.6 (*n* = 2)	44.8 ± 0.3 (*n* = 2)	46.7 ± 1.3 (*n* = 3)
EM.GC80	Not declared	45.2 ± 1.1 (*n* = 3)	45.4 ± 5.0 (*n* = 3)	47.1 ± 0.1 (*n* = 2)	46.9 ± 0.5 (*n* = 2)	47.4 ± 1.1 (*n*= 3)
PELCO50	53	53.4 ± 1.7 (*n* = 3)	55.4 ± 0.4 (*n* = 3)	56.7 ± 0.9 (*n* = 2)	55.7 ± 0.1 (*n* = 2)	54.6 ± 0.9 (*n* = 3)
NISTCaRM	Not declared	50.8 ± 0.6 (*n* = 3)	50.7 ± 0.4 (*n* = 3)	n.d.	51.6 ± 0.4 (*n* = 2)	52.1 ± 1.4 (*n* = 3)

n.d. = not determined.

**Table 7:** Mass concentrations and particle sizes declared on certificate of analysis opposed to those determined in this study.

	Mass Concentration (Average ^2^) [mg L^−1^]		Size—Diameter [nm]	
Test Product	Declared on Certificate of Analysis	Found in Verification Measurements (Section 3.4)	Declared on Certificate of Analysis	Found in Verification Measurements (Section 3.2.2)
EM.GC80	45.2 ^4^	46.4		
NISTCaRM	51.6 ^3^	51.2	60 ^1^	62
PELCO50	53	55.2		

^1^ Nominal diameter. The product provided by NIST was not accompanied by a certificate of analysis. ^2^ Average concentration obtained by laboratories that conducted verification measurements. ^3^ No concentration value was provided for this product. Determination of TEF was conducted using a mass concentration value that was determined in one of the laboratories participating in this study. ^4^ No concentration value was provided on the certificate of analysis for this product. This value was determined as part of the present study.

**Table 8:** Transport efficiencies obtained using three types of nanoparticles.

Material	Average Transport Efficiency Determined by Size Method [%] (*n* = 3)
Gold (reference value)	10.7 ± 0.1
Silver	12.1 ± 0.1 (113% relative vs. gold as reference)
Platinum	16.4 ± 0.3 (153% relative vs. gold as reference)

**Table 9:** Impact of variations of transport efficiency on the particle number concentration.

Monodisperse Test Material		Polydisperse Test Material	
Transport Efficiency (Absolute and Relative Variation)	Particle Number Concentration [Particles mL^−1^] in the Injected Sample and Variation Compared to Reference TE (Bold)	Transport Efficiency (Absolute and Relative Variation)	Particle Number Concentration [Particles mL^−1^] in the Injected Sample and Variation Compared to Reference TE (Bold)
8.56% (−32%)	140,103 (+47%)	6.49% (−38%)	95,600 (+62%)
9.56% (−24%)	125,448 (+31%)	7.49% (−28%)	82,836 (+40%)
10.56% (−16%)	113,568 (+19%)	8.49% (−19%)	73,079 (+24%)
11.56% (−8%)	103,744 (+9%)	9.49% (−9%)	65,379 (+11%)
**12.56** **(Reference)**	**95,484** **(Reference)**	**10.49** **(Reference)**	**59,146** **(Reference)**
13.56% (+8%)	88,443 (−7%)	11.49% (+9%)	53,998 (−9%)
14.56% (+16%)	82,368 (−13%)	12.49% (+19%)	49,675 (−16%)
15.56% (+24%)	77,075 (−19%)	13.49% (+28%)	45,993 (−22%)
16.56% (+32%)	72,420 (−24%)	14.49% (+38%)	42,819 (−28%)

## Data Availability

The data presented in this study are available on request from the corresponding author. The data are not publicly available due to privacy reasons.

## Supplementary Materials

The following supporting information can be downloaded at: https://www.mdpi.com/article/10.3390/nano12040725/s1, SM1: Protocol for the determination of the gold concentration after digestion; SM2: Determination of the gold mass-concentration in gold-nanoparticle suspension test materials by ICP-OES–Detailed procedure; SM3: Protocol for the determination of TES and TEF; SM4: Indicative sample dilution schemes for the determination of TES and TEF; SM5: Responses of ionic gold solutions prepared in ultrapure water and in 1.5 mM sodium citrate; SM6: Scanning Electron Microscopy (SEM) images of the constituent particles of the six test materials; SM7: HAADF-STEM-EDX image of non-gold particles detected in product N8151035 at high magnification; SM8: Particle size distributions of the minimum Feret diameter, the (maximum) Feret diameter, the equivalent circular diameter and the aspect ratio of all tested products; SM9: Comparisons within each laboratory of TES and TEF by AuNP and multiple comparisons of the transport efficiencies calculated using either the size or the frequency approach with different AuNPs; SM10: Stability of shipped samples–Simulation; SM11: Scatter plot of significant correlations between TES and TEF measures for product EM.GC80, LGCQC5050, N8151035, PELCO50; SM12: Determination of transport efficiency following the DMF approach.
