# Supplementary material for: Determination of the Transport Efficiency in spICP-MS Analysis Using Conventional Sample Introduction Systems: An Interlaboratory Comparison Study
Source: Nanomaterials (Basel). 2022 Feb 21;12(4):725. doi: 10.3390/nano12040725 (PMC8875013; doi:10.3390/nano12040725)
Supplement: Supplementary file 1 [file nanomaterials-12-00725-s001.zip › SM1_Digestion Protocol_151121.pdf]

Supplementary Materials – SM1

Protocol for the Determination of the Gold Concentration after Digestion

1. Preparation of Reagents

a) 1L Nitric Acid (1,5%)
23 mL of 65% Nitric acid in ultrapure water

2. Calibration Curve (0 – 20 ppb), Internal Standard Thallium (Tl) at 10 ppb

a) Working Solutions

<u>Ionic Gold Working Solution</u>
Gold (1000 ppm → 10 ppm)
250 µL of Mothersolution (1000 ppm) in 25 mL 1.5% HNO3

<u>Ionic Thallium Solution</u>
Thallium (1000 ppm → 10 ppm)
250 µL of Mothersolution (1000 ppm) in 25 mL 1.5% HNO3

b) Calibration Curve

	**Conc. [ppb]**	**Dilution Factor from Working Solution**	**Volume of Au+ Working Solution in 50 mL HNO3 1,5% [µL]**	**Volume of Thallium Working Solution in 50 mL HNO3 1,5% [µL]**
Cal 0	0		0	50 (=10 ppb)
Cal 5	5	2000	25	50 (=10 ppb)
Cal 10	10	1000	50	50 (=10 ppb)
Cal 15	15	666.6667	75	50 (=10 ppb)
Cal 20	20	500	100	50 (=10 ppb)

3. Sample preparation

Add 100 µL of sample suspensions (concentration should be around 50ppm) into 50 mL Falcon tube
Add 0.15 mL Nitric Acid (65%)
Add 1.5 mL Hydrochloric Acid (37%)
Let sit at RT for 1h
Add 500 µL of Internal standard working solution (10 ppm)
Bring to volume (50 mL) with ULTRAPURE WATER
Dilute 1:10 with Nitric Acid (1.5%)

Blank Sample

Same as above but no gold suspension

4. Recovery Check

Add 500 µL of ionic gold working solution (10 ppm)
Add 0.15 mL Nitric Acid (65%)
Add 1.5 mL Hydrochloric Acid (37%)
Let sit at RT for 1h
Add 500 µL of Internal standard working solution (10 ppm)
Bring to volume (50 mL) with ULTRAPURE WATER
Dilute 1:10 with Nitric Acid (1.5%)
