# Supplementary material for: Determination of the Transport Efficiency in spICP-MS Analysis Using Conventional Sample Introduction Systems: An Interlaboratory Comparison Study
Source: Nanomaterials (Basel). 2022 Feb 21;12(4):725. doi: 10.3390/nano12040725 (PMC8875013; doi:10.3390/nano12040725)
Supplement: Supplementary file 1 [file nanomaterials-12-00725-s001.zip › SM10_Stability shipped samples_151121.pdf]

Supplementary Materials – SM10

Stability of Shipped Samples – Simulation

Undiluted aliquots of the two assessed products were transferred into the same 2 mL vials also used when shipping samples to ILC participants. At defined periods in time (1,2,4,7,9, 17 days), portions were taken from the vials to prepare particle suspensions of 100,000 particles m L^{-1}. The diluted suspensions were then used to determine the transport efficiency by size and by frequency according to the protocol detailed in SM1. Samples were stored at 4 °C in the dark with the exception of the period highlighted in pink in the figure below. In this period samples were left at room temperature to simulate shipping conditions.

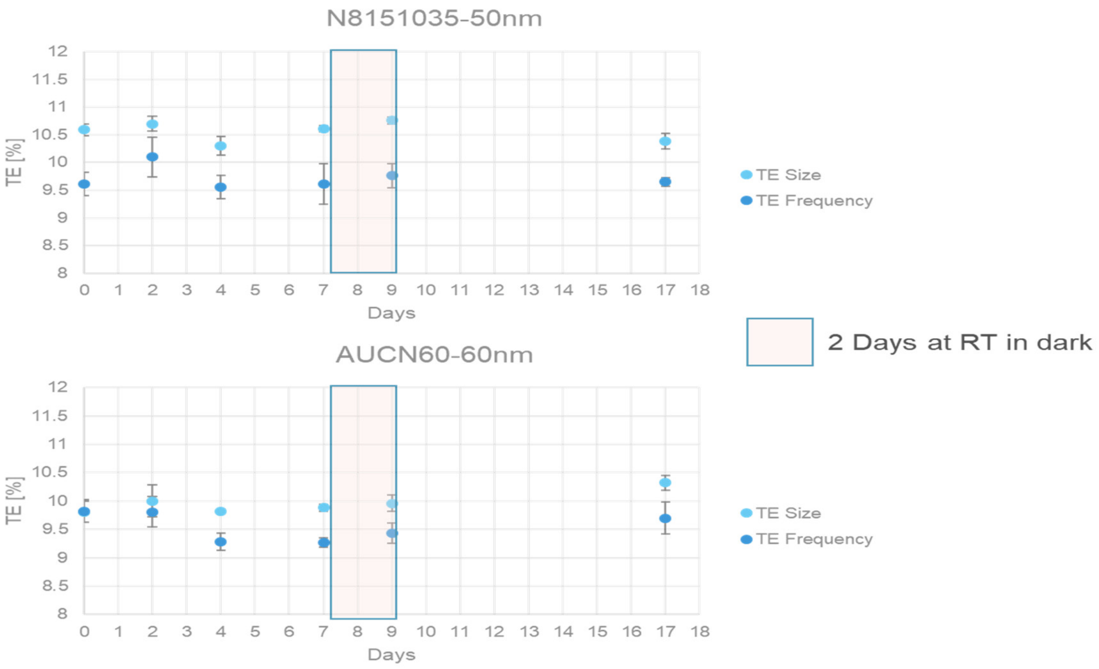

Figure S10. Transport efficiencies (TES and TEF) determined at defined time intervals. Samples were stored at 4 °C except for the period shaded in pink were they were stored at RT
