# Supplementary material for: Determination of the Transport Efficiency in spICP-MS Analysis Using Conventional Sample Introduction Systems: An Interlaboratory Comparison Study
Source: Nanomaterials (Basel). 2022 Feb 21;12(4):725. doi: 10.3390/nano12040725 (PMC8875013; doi:10.3390/nano12040725)
Supplement: Supplementary file 1 [file nanomaterials-12-00725-s001.zip › SM11_Statistics2_071221.pdf]

Supplementary Materials – SM11

Scatter Plot of Significant Correlations Between TES and TEF Measures for Product EM.GC80, LGCQC5050, N8151035, PELCO50

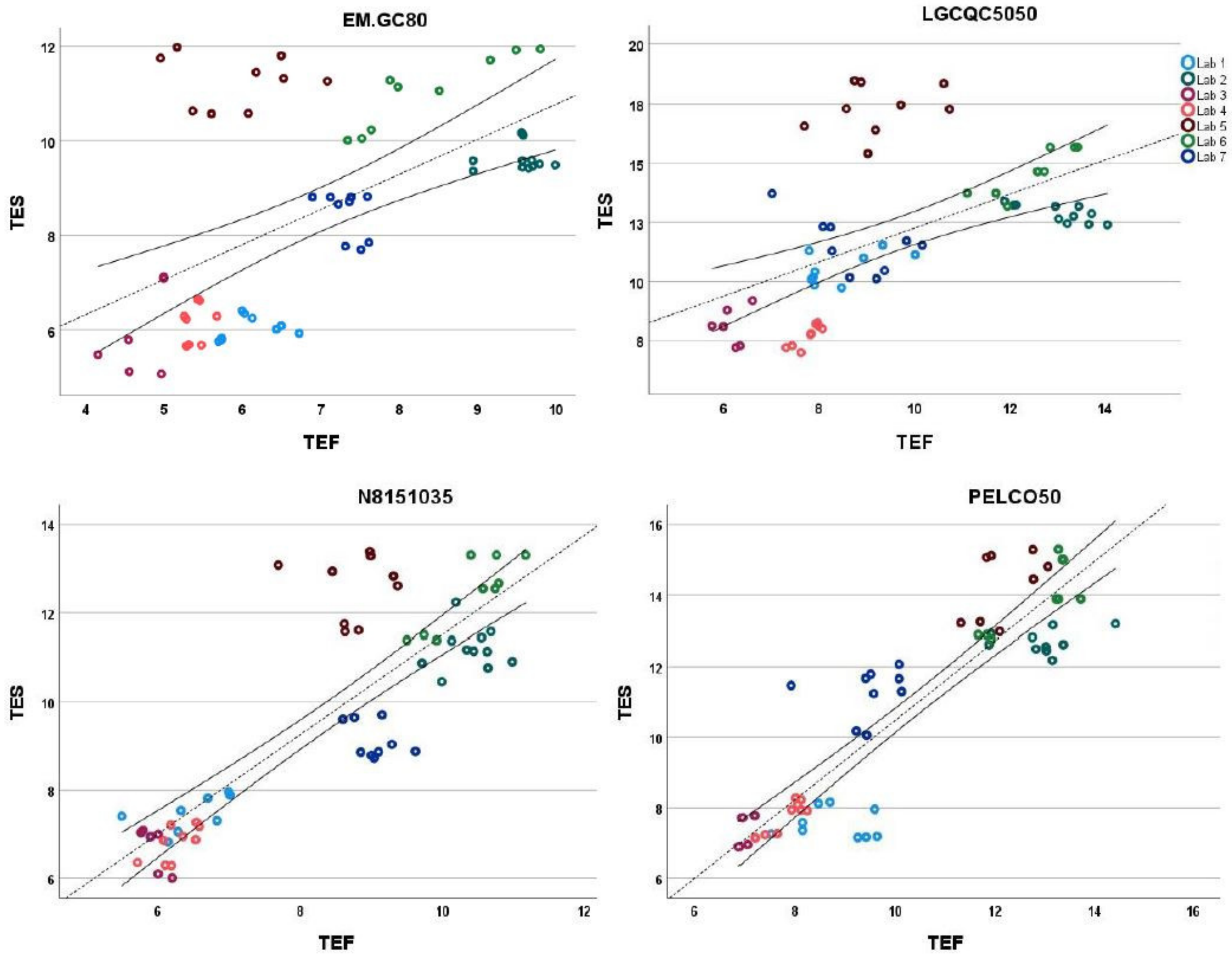

Figure S11. Scatter plot of significant correlations between TES and TEF measures for product EM.GC80, LGCQC5050, N8151035, PELCO50 (rho=0.533, 0.631, 0.777, 0.844, respectively). For the other AuNPs no significant correlations were found (AUCN60: rho=0.153, p=0.236, NISTCaRM: rho=0.290, p=0.053).
