# Supplementary material for: Determination of the Transport Efficiency in spICP-MS Analysis Using Conventional Sample Introduction Systems: An Interlaboratory Comparison Study
Source: Nanomaterials (Basel). 2022 Feb 21;12(4):725. doi: 10.3390/nano12040725 (PMC8875013; doi:10.3390/nano12040725)
Supplement: Supplementary file 1 [file nanomaterials-12-00725-s001.zip › SM12_DMF_071221.pdf]

Supplementary Materials – SM12

Determination of Transport Efficiency Following the DMF Approach

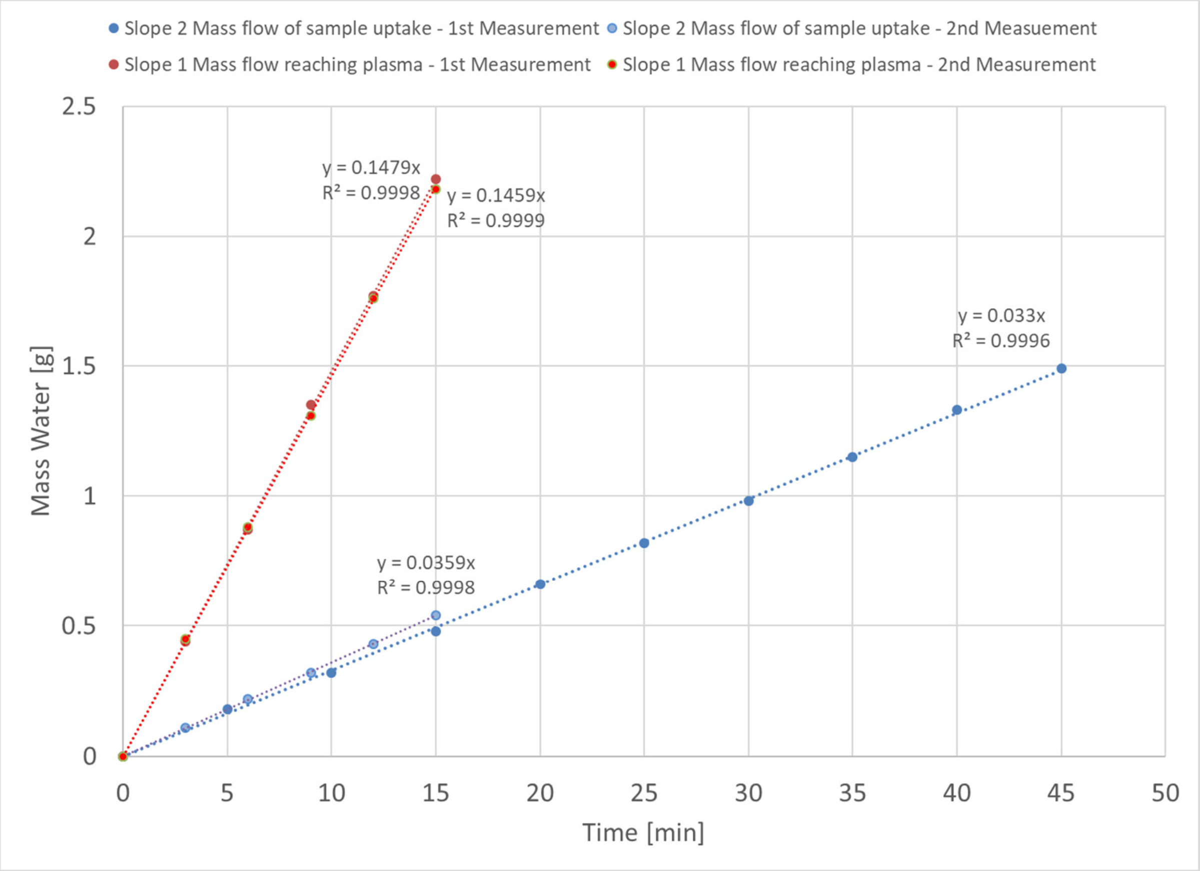

Figure S12. Determination of transport efficiency following the DMF approach. Red lines: Slope 2 from the regression analysis representing mass flow of sample uptake, blue lines: Slope 1 from the regression analysis representing mass flow reaching plasma.
