# Supplementary material for: Determination of the Transport Efficiency in spICP-MS Analysis Using Conventional Sample Introduction Systems: An Interlaboratory Comparison Study
Source: Nanomaterials (Basel). 2022 Feb 21;12(4):725. doi: 10.3390/nano12040725 (PMC8875013; doi:10.3390/nano12040725)
Supplement: Supplementary file 1 [file nanomaterials-12-00725-s001.zip › SM2_ICPOES_251121.pdf]

Supplementary Materials – SM2

Determination of the Gold Mass-Concentration in Gold-Nanoparticle Suspension Test Materials by ICP-OES – Detailed Procedure

Gold-nanoparticle suspensions were thoroughly homogenized with a vortex mixer. Then, 100 μL aliquots were weighed in duplicate in 50 mL PP test tubes and digested at room temperature for one hour with a mixture of 150 μL HNO_3 (69% w/w) and 1500 μL HCl (37% w/w). The volume of the resulting digests was brought to 50 mL with ultrapure water prior to ICP-OES analysis. Blank samples (*i.e.* 100 μL of ultrapure water) and QC samples (*i.e.* 500 μL of 10 mg L^{-1} ionic gold solution) were prepared in duplicate and analyzed following the same procedure for quality control purposes. ICP-OES measurements were performed using an Avio 500 system (Perkin Elmer, Waltham, MA, US) equipped with a 2 mL min^{-1} SeaSpray nebulizer and monitoring Au emission at 242.795 nm. Calibration was achieved using matrix-matched Au standards (0 – 50 – 100 – 150 – 200 μg L^{-1} in 3% v/v HCl and 0.3% v/v HNO_3) prepared from an intermediate ionic gold solution at 10 mg L^{-1}.
