# Supplementary material for: Determination of the Transport Efficiency in spICP-MS Analysis Using Conventional Sample Introduction Systems: An Interlaboratory Comparison Study
Source: Nanomaterials (Basel). 2022 Feb 21;12(4):725. doi: 10.3390/nano12040725 (PMC8875013; doi:10.3390/nano12040725)
Supplement: Supplementary file 1 [file nanomaterials-12-00725-s001.zip › SM3_TES_TEF protocol_151121.pdf]

Supplementary Materials – SM3

Protocol for the Determination of TES and TEF

(Protocol circulated to ILC participants)

The guidance has been optimised for the Perkin Elmer instruments with a dwell-time of 100 µs. For other instruments some of the (dilution) steps possibly require adjustments.

1. Materials

Material identification	Declared Concentration [µg mL^{-1}]	Size – Diameter [nm]
NanoComposix (AUCN60)	54	61 ± 7
Perkin Elmer Ultra-Uniform (N8151035)	0.0124	49.6 ± 2.1
LGC (LGCQC5050)	45.1 ± 1.5	32.7 ± 2.0
BBI Solutions (EM.GC80)	Not declared on certificate. We (JRC) have found 45.2 ± 1.1 ppm.	78.8 ± 8%
Ted Pella - PELCO Highly Uniform (90,050)	53	51 ± 2
NIST Candidate Material	60 (nominal)	50 (nominal)

2. Preparation of dispersant

All ionic gold solutions and NP-suspensions are prepared in 1,5 mM Na-Citrate. Together with the Au-NP suspensions, the parcel contains also a few grams of Citrate (Sigma-Aldrich 71,402).

To prepare 1L of 1.5 mM Na3-Citrate solutions, solve 441.15 mg in 1 L ultrapure water.

3. Sample Flow Measurement

3.1 Make sure the speed of the peristaltic pump is set correctly

3.2 Measure the suction flow rate of the pump by weighing difference with a tube filled with
water over a period of at least 20 min.

3.3 Calculate the flow rate in mL min^{-1} considering the density of the water at 1 g mL^{-1} and note
the values in the attached Excel file (one for each day of analysis).

(If appropriate) set the instrument software for the **determination of Transport Efficiency by Particle Size Method (TES).**

4. Preparation of Ionic Gold Calibration curve

4.1 Stock standard of ionic gold *(1 mg L^{-1} – 1 ppm)*

4.1.1 Transfer 50 µL of 1000 ppm solution into a 50 mL Falcon tube and bring to volume (50 mL) with ultrapure water. (→ by weighing - note exact weights)

4.1.2 Label and store refrigerated (4 °C) at dark.

4.2 Working standards of ionic gold *(0 – 10 µg L^{-1} or ppb)*

According to table 1 pipet the volumes of the stock standard (4.1) into a 50 mL Falcon tube and bring to volume with ultrapure water. Mix thoroughly.

The working standard needs to be prepared freshly each day of analysis.

Table 1. Working standards for ionic Gold

Concentration [µg L^{-1}]	Name	Dilution of Stock standard	Volume of Stock standard [µL]	Final volume [mL]
0 (Blank)	Au^{+} Blank	-	0	50
1	Au^{+} Std 1	1:1000	50	50
3	Au^{+} Std 2	1:333,33	150	50
5	Au^{+} Std 3	1:200	250	50
10	Au^{+} Std 4	1:100	500	50

5. Preparation of NP Gold calibration curve (independent preparations → 3 replicates/material)

Depending on the particle-size and the mass-concentration of the starting product, the dilutions need to be adjusted to reach approximately 100.000 particles mL^{-1}.

Except for the very diluted product distributed by Perkin Elmer (N8151035), all other products need two dilution steps.

NOTE: The reported dilution to 100000 particles mL^{-1} is used by way of example only and can be considered valid for instruments working at µs dwell time ranges. For ms dwell-time ranges, a higher dilution might be required.

5.1 First dilution Step

5.1.1 Let the monodisperse spherical gold nanoparticle suspension reach room temperature and bath sonicate for 300s at the maximum power.

5.1.2 Vortex stir for 30 s

5.1.3 Prepare 1st dilution steps (**by weighing**) as described in the table

	AUCN60	N8151035	LGCQC5050	EM.GC80	Ted Pella
Size [nm]	61	49.6	32.7	78.8	51
Conc. [µg/mL]	54	0.0124	45.1	45.2	53
Dilution [1:X]	200	100	1000	200	200
Volume of undiluted product in X mL 1.5mM Na3-Citrate	50 µL in 10 mL Citrate	50 µL in 5 mL Citrate	50 µL in 50 mL Citrate	50 µL in 10 mL Citrate	50 µL in 10 mL Citrate

5.1.4 If not immediately used, label and store in refrigerator (4 °C) at dark.

5.1.5 If properly stored, the stock standard suspension can be considered stable for at least one day.

5.2 Working standard gold nanoparticles

(Target concentration ca 100.000 particles mL^{-1}]

5.2.1 Prepare the following second dilutions (**by weighing**) to reach the desired 100.000 particles mL^{-1}.

	AUCN60	N8151035	LGCQC5050	EM.GC80	Ted Pella
Size [nm]	61	49.6	32.7	78.8	51
Conc. [µg/mL]	54	0.0124	45.1	45.2	53
Dilution [1:X]	1177	No 2nd step required	1276	457	1976
Volume of 1st step dilution in X mL 1.5 mM Citrate	42.5 µL in 50 mL 1.5 mM Citrate		39.2 µL in 50 mL 1.5 mM Citrate	109.4 µL in 50 mL 1.5 mM Citrate	25.3 µL in 50 mL 1.5 mM Citrate

For details of calculation, please see Annex 1 and excel file provided via email

Please, note the value of the theoretical particle number concentration (part/mL) in the attached Excel file for all the Gold Standards

5.2.2 Vortex stir for 30 s

The Transport Efficiency (TE) is automatically determined by the instrument's software. Please note the values of both Transport Efficiency and Particle number (events) per scan time in the attached Excel file.

Determination of transport efficiency by particle frequency method (TEF)

The data required to (manually) calculate the **transport efficiency by frequency** can be extracted from the same data file acquired for the determination of TE by size:

Sample	Analyte	Most Freq. Size (nm)	Mean Size (nm)	No. of Peaks	Mean Inten. (counts)	Part. Conc. (parts/mL)
Blank	Au 197					
STD1	Au 197					
STD2	Au 197					
STD3	Au 197					
STD4	Au 197					
Blank	Au 197					
STD1	Au 197			1893	122.51	

Please use the following equations:

a) Detected Particle number per mL=Particle number (events) per scan time (min)/flow rate (mL* min^{-1})

b) TEF= Detected Particle number per mL/Theoretic Particle number per mL

Calculation of TEF is done automatically by filling the attached Excel file with the required values.

SAMPLE SEQUENCE (one for each day of test)

Ionic Calibration

Au^+ Blank (ultrapure water)

Au^+ Std 1

Au^+ Std 2

Au^+ Std 3

Au^+ Std 4

Samples

Np-Au Blank (1.5 mM Na3-Citrate)

AUCN60-1 (Replicate 1)

AUCN60-1 as sample for QC

Np-Au Blank (1.5 mM Na3-Citrate)
AUCN60-2 (Replicate 2)
AUCN60-2 as sample for QC
Np-Au Blank (1.5 mM Na3-Citrate)
AUCN60-2 (Replicate 3)
AUCN60-2 as sample for QC

Np-Au Blank (1.5 mM Na3-Citrate)
N8151035-1 (Replicate 1)
N8151035-1 as sample for QC

Np-Au Blank (1.5 mM Na3-Citrate)
N8151035-2 (Replicate 2)
N8151035-2 as sample for QC

Np-Au Blank (1.5 mM Na3-Citrate)
N8151035-2 (Replicate 3)
N8151035-2 as sample for QC

… (same pattern for other products)
