# Supplementary material for: Determination of the Transport Efficiency in spICP-MS Analysis Using Conventional Sample Introduction Systems: An Interlaboratory Comparison Study
Source: Nanomaterials (Basel). 2022 Feb 21;12(4):725. doi: 10.3390/nano12040725 (PMC8875013; doi:10.3390/nano12040725)
Supplement: Supplementary file 1 [file nanomaterials-12-00725-s001.zip › SM4_Indicative dilution scheme_151121.pdf]

Supplementary Materials – SM4

Indicative Sample Dilution Schemes for the Determination of TES and TEF

a) Instruments with micro-second dwell-times

Table 4. a. Required dilution of tested products to reach 100000 particles mL^{-1}. Instruments with micro-second dwell-times.

	AUCN60	N8151035	LGCQC5050	EM.GC80	PELCO50	NISTCaRM
Size [nm]	61	49.6	32.7	78.8	51	60
Conc. [µg/mL]	54	0.0124	45.1	46.4	53	51.2
First Dilution Step						
Dilution [1:X]	200	100	1000	200	200	200
Volume of undiluted product in X mL 1.5 mM Na3-Citrate	50 µL in 10 mL Citrate	50 µL in 5 mL Citrate	50 µL in 50 mL Citrate	50 µL in 10 mL Citrate	50 µL in 10 mL Citrate	50 µL in 10 mL Citrate
Second Dilution Step						
Dilution [1:X]	1177	No 2nd step required	1276	457	1976	1177
Volume of 1st step dilution in X mL 1.5 mM Citrate	42.5 µL in 50 mL 1.5 mM Citrate		39.2 µL in 50 mL 1.5 mM Citrate	109.4 µL in 50 mL 1.5 mM Citrate	25.3 µL in 50 mL 1.5 mM Citrate	42.5 µL in 50 mL 1.5 mM Citrate

b) Instruments with milli-second dwell-times

Table S4b. Required dilution of tested products to reach 100000 particles mL^{-1}. Instruments with milli-second dwell-times

	AUCN60	N8151035	LGCQC5050	EM.GC80	PELCO50	NISTCaRM
First Dilution Step						
Dilution [1:X]	1000	250	1000	1000	1000	1000
Volume of undiluted product in X mL 1.5 mM Na-Citrate	10 µL in 10 mL Citrate	40 µL in 10 mL Citrate	10 µL in 10 mL Citrate	10 µL in 10 mL Citrate	10 µL in 10 mL Citrate	10 µL in 10 mL Citrate
Second Dilution Step						
Dilution [1:X]	10	No 2nd step required	10	10	10	10
Volume of 1st step dilution in X mL 1.5 mM Citrate	1 mL in 10 mL 1.5 mM Citrate		1 mL in 10 mL 1.5 mM Citrate	1 mL in 10 mL 1.5 mM Citrate	1 mL in 10 mL 1.5 mM Citrate	1 mL in 10 mL 1.5 mM Citrate
Third Dilution Step						
Dilution [1:X]	10		10	10	10	10
Volume of 2nd step dilution in X mL 1.5 mM Citrate	1 mL in 10 mL 1.5 mM Citrate		1 mL in 10 mL 1.5 mM Citrate	1 mL in 10 mL 1.5 mM Citrate	1 mL in 10 mL 1.5 mM Citrate	1 mL in 10 mL 1.5 mM Citrate
Fourth Dilution Step						

Dilution [1:X]	10		10	10	10	10
Volume of 3rd step dilution in X mL 1.5 mM Citrate	1 mL in 10 mL 1.5 mM Citrate		1 mL in 10 mL 1.5 mM Citrate	1 mL in 10 mL 1.5 mM Citrate	1 mL in 10 mL 1.5 mM Citrate	1 mL in 10 mL 1.5 mM Citrate
