# Supplementary material for: Determination of the Transport Efficiency in spICP-MS Analysis Using Conventional Sample Introduction Systems: An Interlaboratory Comparison Study
Source: Nanomaterials (Basel). 2022 Feb 21;12(4):725. doi: 10.3390/nano12040725 (PMC8875013; doi:10.3390/nano12040725)
Supplement: Supplementary file 1 [file nanomaterials-12-00725-s001.zip › SM5_Responses ionic Au_301121.pdf]

Supplementary Materials – SM5

Responses of Ionic Gold Solutions Prepared in Ultrapure Water and in 1.5 mM Sodium Citrate

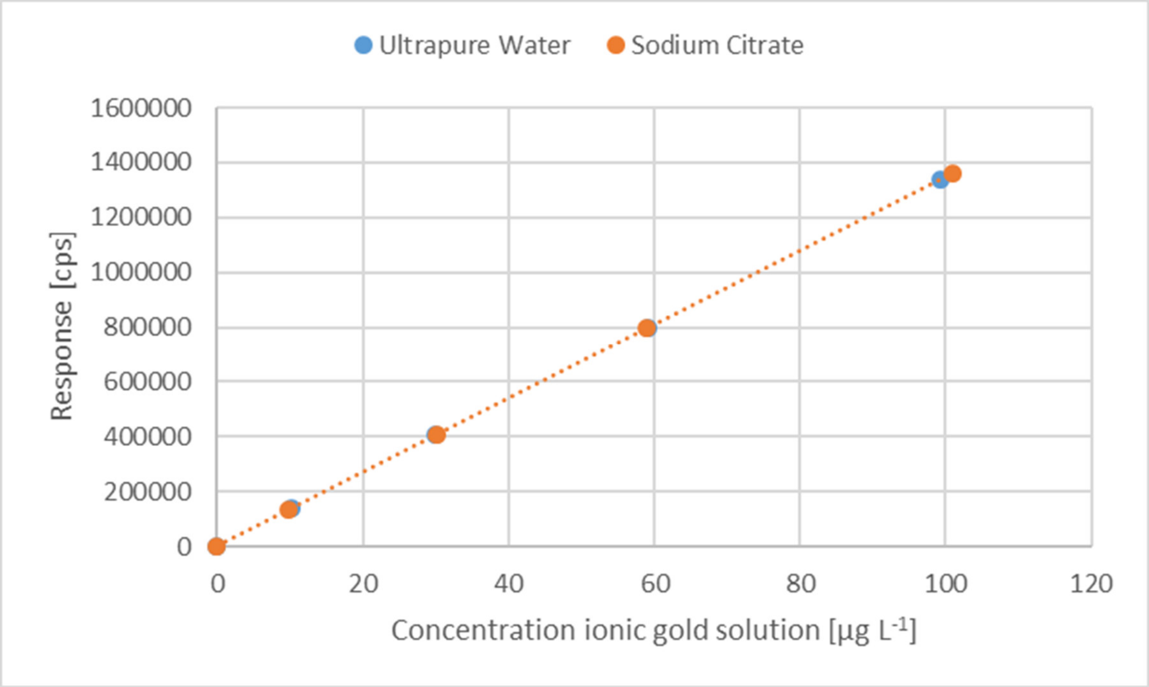

Figure S5. Responses of ionic gold solutions prepared in ultrapure water and in 1.5 mM sodium citrate
