# Supplementary material for: Determination of the Transport Efficiency in spICP-MS Analysis Using Conventional Sample Introduction Systems: An Interlaboratory Comparison Study
Source: Nanomaterials (Basel). 2022 Feb 21;12(4):725. doi: 10.3390/nano12040725 (PMC8875013; doi:10.3390/nano12040725)
Supplement: Supplementary file 1 [file nanomaterials-12-00725-s001.zip › SM6_SEM Images_161121.pdf]

Supplementary Materials – SM6

Scanning Electron Microscopy (SEM) Images of the Constituent Particles of the Six Test Materials

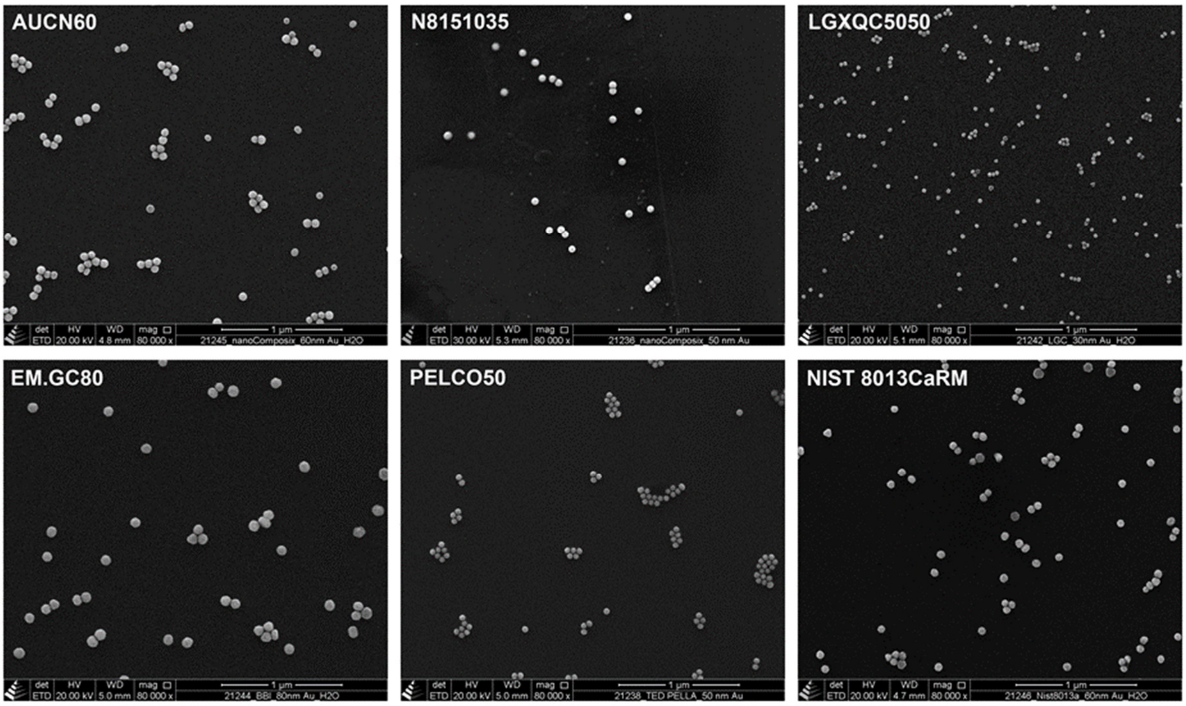

Figure S6. SEM images resulting from the image analysis of the constituent particles of the six test materials.
