# Supplementary material for: Determination of the Transport Efficiency in spICP-MS Analysis Using Conventional Sample Introduction Systems: An Interlaboratory Comparison Study
Source: Nanomaterials (Basel). 2022 Feb 21;12(4):725. doi: 10.3390/nano12040725 (PMC8875013; doi:10.3390/nano12040725)
Supplement: Supplementary file 1 [file nanomaterials-12-00725-s001.zip › SM7_EDX_161121.pdf]

Supplementary Materials – SM7

HAADF-STEM-EDX Image of Non-Gold Particles Detected in Product N8151035 at High Magnification

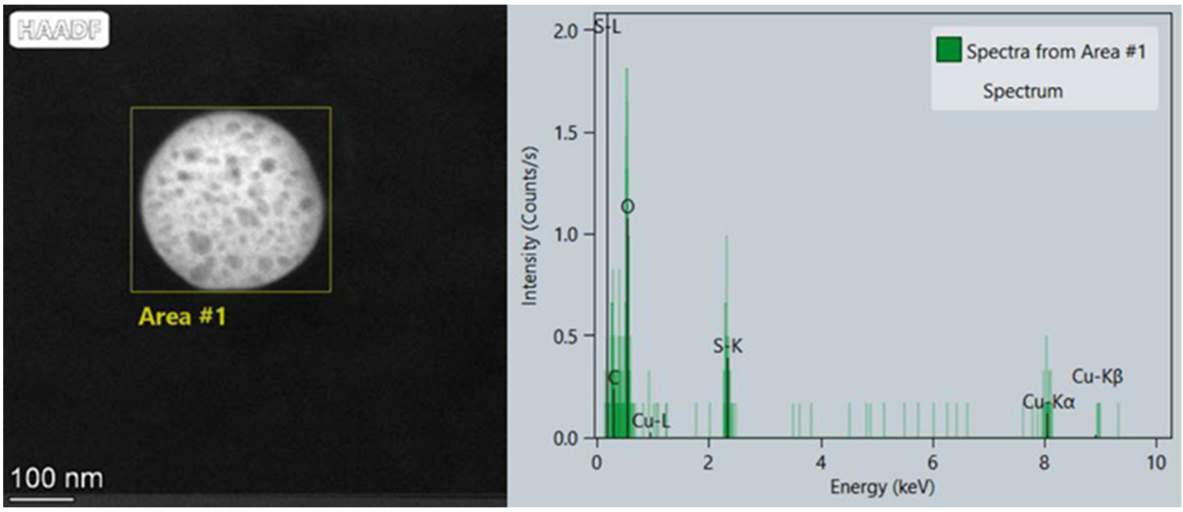

Figure S7. HAADF-STEM-EDX image of non-gold particles detected in product N8151035 at high magnification (x12600) (left) and corresponding EDX spectrum (right) showing the elemental composition for Area 1
