# Supplementary material for: Determination of the Transport Efficiency in spICP-MS Analysis Using Conventional Sample Introduction Systems: An Interlaboratory Comparison Study
Source: Nanomaterials (Basel). 2022 Feb 21;12(4):725. doi: 10.3390/nano12040725 (PMC8875013; doi:10.3390/nano12040725)
Supplement: Supplementary file 1 [file nanomaterials-12-00725-s001.zip › SM8_TEM distributions_161121.pdf]

# Supplementary Materials – SM8

## Particle Size Distributions of the Minimum Feret Diameter, the (Maximum) Feret diameter, the Equivalent Circular Diameter and the Aspect Ratio of all tested Products

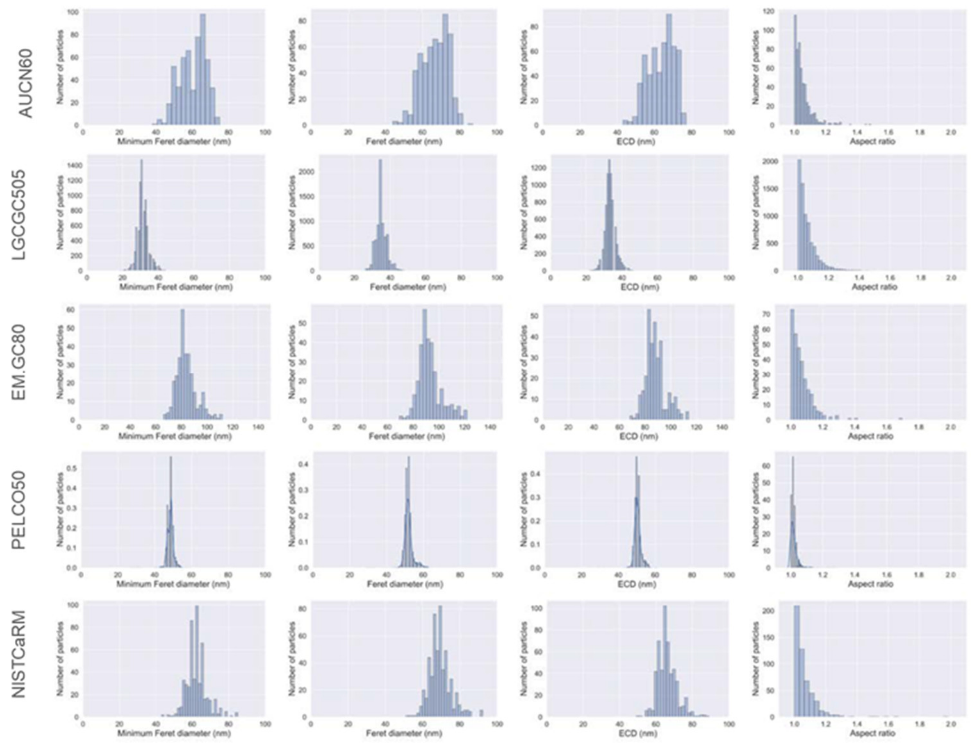


**Figure S8a.** Particle size distributions of the minimum Feret diameter, the (maximum) Feret diameter, the equivalent circular diameter and the aspect ratio of all tested products except test material N8151035

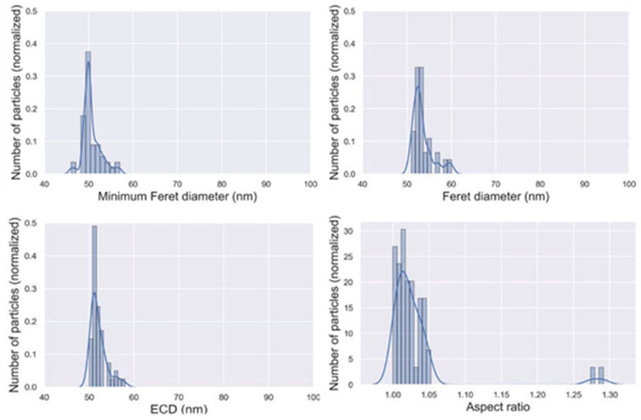


**Figure S8b.** Particle size distributions of the minimum Feret diameter, the (maximum) Feret diameter, the equivalent circular diameter and the aspect ratio of test material N8151035
