# Supplementary material for: Determination of the Transport Efficiency in spICP-MS Analysis Using Conventional Sample Introduction Systems: An Interlaboratory Comparison Study
Source: Nanomaterials (Basel). 2022 Feb 21;12(4):725. doi: 10.3390/nano12040725 (PMC8875013; doi:10.3390/nano12040725)
Supplement: Supplementary file 1 [file nanomaterials-12-00725-s001.zip › SM9_Statistics1_071221.pdf]

Supplementary Materials – SM9

Comparisons within each Laboratory of TES and TEF by AuNP and Multiple Comparisons of the Transport Efficiencies Calculated using Either the size or the Frequency Approach with different AuNPs

Table S9. Comparisons within each laboratory of TES and TEF by AuNP and multiple comparisons of the transport efficiencies calculated using either the size or the frequency approach with different AuNPs. TES and TEF are shown as means and standard deviations (SD).

Laboratory	AuNPs	**TES %** Mean (SD)	Multiple comparisons	**TEF %** Mean (SD)	Multiple comparisons
**Lab 1**	AUCN60	7.85 (0.41)°°	EM.GC80 vs AUCN60**<br>EM.GC80 vs PELCO50*<br>EM.GC80 vs LGCQC5050***<br>NISTCaRM vs AUCN60*<br>NISTCaRM vs LGCQC5050***	6.11 (0.51)	EM.GC80 vs LGCQC5050***<br>EM.GC80 vs PELCO50***<br>AUCN60 vs LGCQC5050**<br>AUCN60 vs PELCO50***<br>N8151035 vs LGCQC5050*<br>N8151035 vs PELCO50**
	EM.GC80	6.05 (0.24)		6.10 (0.37)	
	LGCQC5050	10.58 (0.67)°°		8.46 (0.80)	
	N8151035	7.42 (0.42)°°		6.41 (0.52)	
	NISTCaRM	6.36 (0.35)		6.66 (0.36)	
	PELCO50	7.55 (0.42)°°		8.76 (0.76)	
**Lab 2**	AUCN60	11.45 (0.51)°°	EM.GC80 vs PELCO50***<br>EM.GC80 vs LGCQC5050***<br>N8151035 vs PELCO50*<br>N8151035 vs LGCQC5050**<br>AUCN60 vs LGCQC5050*	10.04 (0.36)	EM.GC80 vs PELCO50***<br>EM.GC80 vs LGCQC5050***<br>NISTCaRM vs LGCQC5050*<br>AUCN60 vs LGCQC5050**<br>AUCN60 vs PELCO50**
	EM.GC80	9.61 (0.27)		9.54 (0.32)	
	LGCQC5050	12.89 (0.37)		13.05 (0.72)	
	N8151035	11.19 (0.47)°°		10.39 (0.36)	
	NISTCaRM	11.11 (0.08)		9.78 (0.08)	
	PELCO50	12.70 (0.32)		12.96 (0.72)	
**Lab 3**	AUCN60	6.16 (1.23)	NISTCaRM vs LGCQC5050**<br>EM.GC80 vs LGCQC5050*	6.30 (0.42)	EM.GC80 vs AUCN60*<br>EM.GC80 vs PELCO50***<br>NISTCaRM vs PELCO50**
	EM.GC80	5.95 (0.94)°		4.70 (0.34)	
	LGCQC5050	8.11 (0.79)°		6.18 (0.30)	
	N8151035	6.70 (0.50)		5.94 (0.16)	
	NISTCaRM	5.46 (0.46)		4.74 (0.60)	
	PELCO50	7.34 (0.47)		7.01 (0.13)	
**Lab 4**	AUCN60	6.46 (0.40)°	EM.GC80 vs LGCQC5050***<br>EM.GC80 vs PELCO50***<br>AUCN60 vs LGCQC5050**<br>AUCN60 vs PELCO50**	6.17 (0.25)	EM.GC80 vs LGCQC5050***<br>EM.GC80 vs PELCO50***<br>AUCN60 vs LGCQC5050*<br>AUCN60 vs PELCO50*<br>N8151035 vs PELCO50*
	EM.GC80	6.20 (0.42)°°		5.40 (0.13)	
	LGCQC5050	7.73 (0.47)		7.78 (0.27)	
	N8151035	6.81 (0.40)°°		6.25 (0.28)	
	NISTCaRM	-		-	
	PELCO50	7.78 (0.44)		7.85 (0.36)	
**Lab 5**	AUCN60	12.71 (0.72)°°	EM.GC80 vs LGCQC5050***<br>EM.GC80 vs PELCO50**<br>N8151035 vs LGCQC5050**<br>NISTCaRM vs LGCQC5050**<br>AUCN60 vs LGCQC5050*	4.14 (1.03)	AUCN60 vs N8151035**<br>AUCN60 vs LGCQC5050**<br>AUCN60 vs PELCO50***<br>NISTCaRM vs LGCQC5050*<br>NISTCaRM vs PELCO50***<br>EM.GC80 vs PELCO50**
	EM.GC80	11.26 (0.55)°°		5.93 (0.71)	
	LGCQC5050	17.31 (1.05)°°		9.25 (0.97)	
	N8151035	12.56 (0.73)°°		8.76 (0.51)	
	NISTCaRM	12.64 (0.73)°°		5.34 (0.99)	
	PELCO50	14.29 (0.91)°°		12.26 (0.61)	
**Lab 6**	AUCN60	11.13 (0.78)°°	EM.GC80 vs LGCQC5050***<br>EM.GC80 vs PELCO50***<br>AUCN60 vs LGCQC5050***<br>AUCN60 vs PELCO50**	7.41 (0.90)	AUCN60 vs N8151035*<br>AUCN60 vs LGCQC5050***<br>AUCN60 vs PELCO50***<br>NISTCaRM vs LGCQC5050**<br>NISTCaRM vs PELCO50**<br>EM.GC80 vs LGCQC5050**<br>EM.GC80 vs PELCO50**
	EM.GC80	11.04 (0.77)°°		8.37 (0.91)	
	LGCQC5050	14.63 (0.93)°°		12.48 (0.76)	
	N8151035	12.44 (0.82)°°		10.4 (0.56)	
	NISTCaRM	12.44 (0.82)°°		8.34 (0.69)	
	PELCO50	13.95 (0.98)°°		12.86 (0.80)	

**Lab 7**	AUCN60<br>EM.GC80<br>LGCQC5050<br>N8151035<br>NISTCaRM<br>PELCO50	8.81 (0.54)<br>8.44 (0.50)°°<br>11.51 (1.18)°°<br>9.13 (0.41)<br>9.34 (0.57)°°<br>11.27 (0.70)°°	EM.GC80 vs LGCQC5050***<br>EM.GC80 vs PELCO50***<br>AUCN60 vs LGCQC5050**<br>AUCN60 vs PELCO50**<br>N8151035 vs LGCQC5050*	6.72 (2.84)<br>7.33 (0.23)<br>8.77 (0.98)<br>9.05 (0.3)<br>7.55 (0.65)<br>9.48 (0.68)	EM.GC80 vs LGCQC5050**<br>EM.GC80 vs PELCO50***<br>NISTCaRM vs N8151035*<br>NISTCaRM vs PELCO50**<br>AUCN60 vs PELCO50*

°p<0.05, °°p<0.01 TES vs TEF

*p<0.05, **p<0.01, ***p<0.001 multiple comparisons of the TEs by AuNP (using either the TES or TEF approach)
